# Transcriptomic insight into salinomycin mechanisms in breast cancer cell lines: synergistic effects with dasatinib and induction of estrogen receptor β

**DOI:** 10.1186/s12885-020-07134-3

**Published:** 2020-07-16

**Authors:** Vanessa Bellat, Alice Verchère, Sally A. Ashe, Benedict Law

**Affiliations:** 1 Molecular Imaging Innovations Institute, Department of Radiology, Weill Cornell Medicine, New York, NY USA; 2 Department of Biochemistry, Weill Cornell Medicine, New York, NY USA; 3 Lead contact, New York, USA

**Keywords:** Synergistic drug combination, Transcriptome and proteomic analysis, Cell signaling pathway, Estrogen receptor β (ESR2), Triple negative breast cancer (TNBC)

## Abstract

**Background:**

Tumors are heterogeneous in nature, composed of different cell populations with various mutations and/or phenotypes. Using a single drug to encounter cancer progression is generally ineffective. To improve the treatment outcome, multiple drugs of distinctive mechanisms but complementary anticancer activities (combination therapy) are often used to enhance antitumor efficacy and minimize the risk of acquiring drug resistance. We report here the synergistic effects of salinomycin (a polyether antibiotic) and dasatinib (a Src kinase inhibitor).

**Methods:**

Functionally, both drugs induce cell cycle arrest, intracellular reactive oxygen species (iROS) production, and apoptosis. We rationalized that an overlapping of the drug activities should offer an enhanced anticancer effect, either through vertical inhibition of the Src-STAT3 axis or horizontal suppression of multiple pathways. We determined the toxicity induced by the drug combination and studied the kinetics of iROS production by fluorescence imaging and flow cytometry. Using genomic and proteomic techniques, including RNA-sequencing (RNA-seq), reverse transcription-quantitative polymerase chain reaction (RT-qPCR), and Western Blot, we subsequently identified the responsible pathways that contributed to the synergistic effects of the drug combination.

**Results:**

Compared to either drug alone, the drug combination showed enhanced potency against MDA-MB-468, MDA-MB-231, and MCF-7 human breast cancer (BC) cell lines and tumor spheroids. The drug combination induces both iROS generation and apoptosis in a time-dependent manner, following a 2-step kinetic profile. RNA-seq data revealed that the drug combination exhibited synergism through horizontal suppression of multiple pathways, possibly through a promotion of cell cycle arrest at the G1/S phase via the estrogen-mediated S-phase entry pathway, and partially via the BRCA1 and DNA damage response pathway.

**Conclusion:**

Transcriptomic analyses revealed for the first time, that the estrogen-mediated S-phase entry pathway partially contributed to the synergistic effect of the drug combination. More importantly, our studies led to the discoveries of new potential therapeutic targets, such as E2F2, as well as a novel drug-induced targeting of estrogen receptor β (ESR2) approach for triple-negative breast cancer treatment, currently lacking of targeted therapies.

## Background

Advances in screening, early diagnosis, and treatment have significantly reduced the mortality rate of breast cancer (BC) for the past 20 years. However, the 5-years survival rate of patients with late-stage metastatic BC remains low (less than 30%) [1]. Chemotherapeutic agents and targeted therapies are the current backbone of medical management. Patients do not always respond to these treatments, as tumors can be intrinsically (de novo) resistant to the drugs. Furthermore, patients who initially responds to the treatments will likely acquire resistance over time, resulting in treatment failure or disease recurrence. To improve clinical outcomes, multiple chemotherapeutic agents (combination therapy) with distinctive drug mechanisms are used for BC treatments [2, 3]. However, most clinically used drug combination regimens only increase overall response rate and prolong progression-free survival, but showed limited success for improving the patient’s survival [4].

**Fig. 1:**
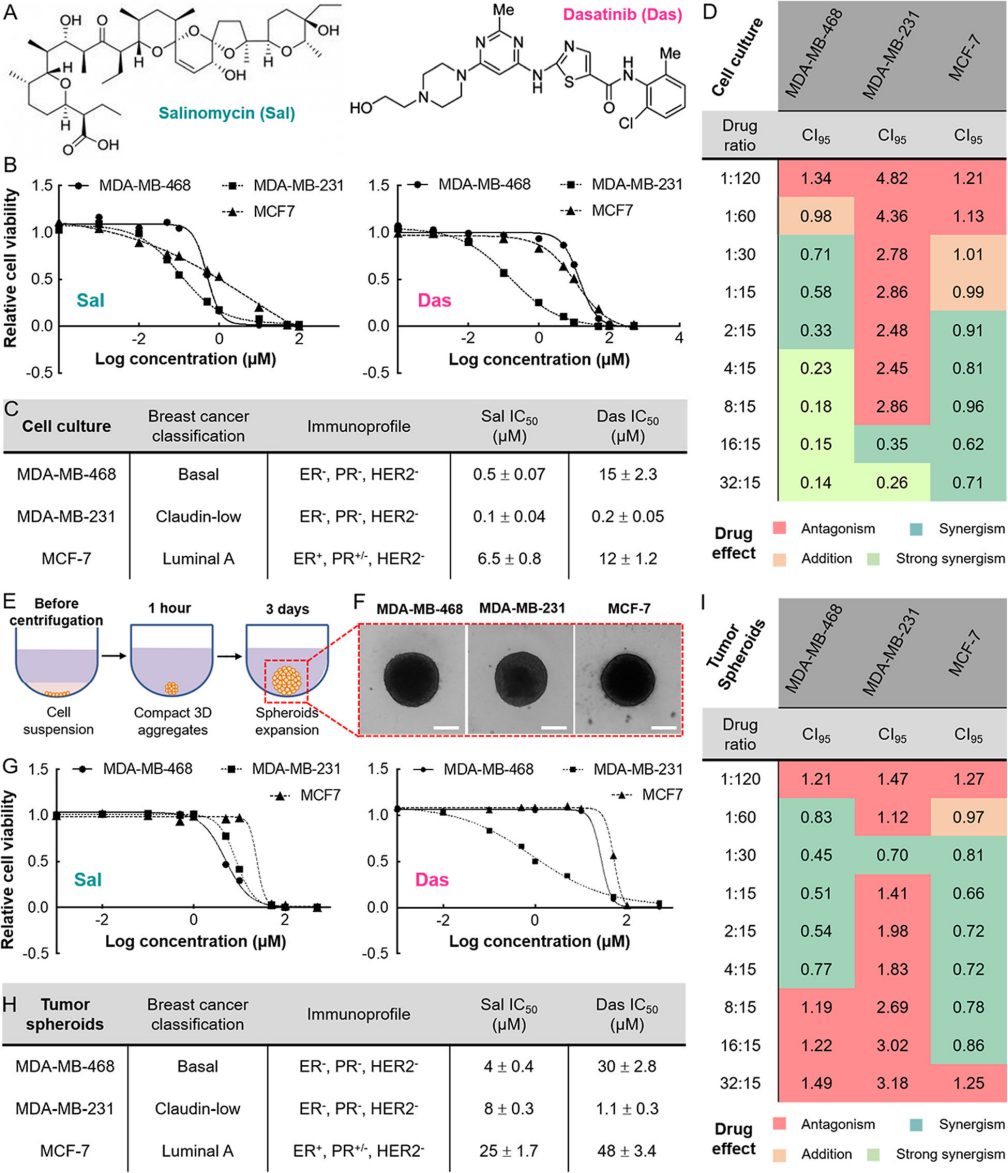
Evaluation of the cytotoxicity of salinomycin (Sal) and dasatinib (Das) as single drugs or a 2-drug combination on MDA-MB-468, MDA-MB-231, and MCF-7 cell lines (monolayer cell culture system) and tumor spheroids. **a** Chemical structures of the drugs. **b** A comparison of the potencies of individual drugs. To measure cell viability, different human BC cell lines, cultured in monolayers, were incubated with the drugs at various concentrations for 72 h. The results were fit into sigmoidal dose response curves for calculating the IC_50_ values. **c** A table summarizing the specific IC_50_ values of both Sal and Das. Sal was more potent than Das regardless of the cell line tested. **d** A table summarizing the synergism of the same drug combination but different applied drug ratios of Sal and Das for treating various BC cell lines. Drug combinations had a stronger synergistic effect on MDA-MB-468, as shown by the lower CI_95_ values. The CI_95_ values were determined using the previously described Chou-Talalay method [6]. Note that CI_95_ represents the specific CI value where there is a 95% cell growth inhibition. **e** A schematic diagram showing the method for preparing tumor spheroids. **f** Representative microscopic images of the MDA-MB-468, MDA-MB-231, and MCF-7 spheroids. Scale bar is 200 μm. **g** The cytotoxic effect of the drugs alone. The spheroids were treated with drugs at various concentrations for 72 h. The results from the viability assays were fit into sigmoidal dose response curves for determining the IC_50_ values. **h** A table summarizing the specific IC_50_ values of Sal and Das tested on different tumor spheroids. **i** A comparison of the synergism of different drug combination regimens, applied concurrently at different drug ratios, for eradicating the spheroids. All the experiments were independently performed in triplicate

It has now become clear that tumors are heterogeneous in nature, composed of different cell populations with various mutations and/or phenotypes. Chemotherapeutic agents primarily eliminate the proliferating cells in tumors but can leave behind a small population of quiescent cancer stem cells (CSCs) that are intrinsically resistant to chemotherapy. These residual CSCs, which have metastatic potential, can remodel tumors to become more drug resistant [5]. Salinomycin (Sal) is an antibiotic isolated from *Streptomyces albus* that has been used as an anticoccidial agent in the poultry industries for many years (Fig. 1a). In recent years, the drug has been shown to have anti-CSCs properties [7]. Among 16,000 compounds screened as potential toxic substances against breast CSCs, Sal was able to selectively reduce the proportion of epithelial cancer stem cells by more than 100-folds compared to paclitaxel, a drug that is commonly used as a chemotherapeutic agent for BC. Early studies showed that Sal induced apoptosis by disrupting the balance of sodium and potassium ions across the mitochondrial membranes [8, 9]. The drug induced intracellular reactive oxygen species (iROS) production, and subsequently mediated autophagy via activation of the JNK/MAPK pathway [10]. Sal also has been shown to suppress the highly conserved embryonic developmental signaling pathways, including the STAT3, Notch, Wnt/β-catenin, and hedgehog pathways [11, 12]. The drug inhibited proliferation, induced apoptosis, and reduced the metastatic potential of CSCs and other cancer cells [13–18]. When used in a drug combination, Sal increased DNA damage in BC cells treated with doxorubicin (Dox) or etoposide [19, 20]. It also enhanced the effects of paclitaxel to induce apoptosis and prevent G2 arrest [21].

Dasatinib (Das) is a Src kinase inhibitor, and has been approved for the treatment of chronic myelogenous leukemia and acute lymphoblastic leukemia (Fig. 1a). However, its role in treating BC is uncertain. BC patients only showed limited response to monotherapy of Das [22]. Preclinical studies showed that Das inhibited BC cells by modulating epidermal growth factor receptor (EGFR) signaling [23]. Src is an upstream regulator of the STAT3, PI3K, and Ras/MAPK pathways [24]. Using Das to inhibit Src can suppress BC cell proliferation, migration, invasion, and angiogenesis [25]. Recently, Das was also shown to display anti-CSC effects. The drug reduced the percentage of aldehyde dehydrogenase 1 (ALDH1)-positive CSC populations in triple negative BC (TNBC) cell lines [26]. In this paper, we investigated the feasibility of using a combination of Sal and Das (S + D) to counter BC. Functionally, both drugs induce cell cycle arrest, iROS production, and apoptosis. We rationalized that an overlapping of the drug activities should offer an enhanced anticancer effect, either through vertical inhibition of the Src-STAT3 axis [17, 27] or horizontal suppression of multiple pathways. We studied the kinetics of iROS, and the toxicity induced by the drug combination. Using RNA-seq, we subsequently identified the responsible pathways, including the estrogen-mediated S-phase entry pathway, that partially contributed to the synergistic effects of the drug combination. These studies led to discoveries of potential therapeutic targets, such as E2F2 as well as a novel drug-induced targeting of estrogen receptor β (ESR2) approach, which were also described herein.

## Methods

### Chemicals and supplies

All reagents and resources used for this study, as well as their source and identifier, are listed in **Table****S1****.**

### Cell culture

All the human BC cell lines were purchased directly from ATCC (Manassas, VA) in 2016. All the cell lines were cytogenetic analyzed by ATCC. Upon arrival, the cells were cultured according to the ATCC’s instructions to prepare stocks for long-term cryopreservation. Prior to perform the in vitro experiments, the cells were tested for mycoplasma contamination using the MycoAlert mycoplasma detection kit (Lonza, Basel, Switzerland). To avoid genetic drift that may affect the results and reproducibility, cells were not cultured for more than 6 months. To limit any undesired fluorescence interaction during flow cytometry analysis and fluorescence imaging, cells were cultured in the absence of phenol red.

### Determination of the drugs potency

To assess the cytotoxicity of the drugs alone or in combination, cells (5 × 10^3^ cells/well) were seeded on a 96-well flat bottom plate overnight, and then treated with various concentrations of the drugs for 72 h. After washing the cells 3 times with PBS, CellTiter-Glo 2D reagent (Promega, Madison, WI) (50 μL) was added to each well. The luminescence was recorded using a microplate reader (Tecan US Inc., Morrisville, NC). The dose-response curves were plotted using GraphPad Prism 6.0 software. All data were normalized to the values obtained with untreated control cells. The half maximal inhibitory concentrations (IC_50_) were calculated by fitting the data into a sigmoidal curve. The cytotoxicity of the different drug treatments was also evaluated by Trypan Blue Exclusion assay. After incubation with the drug alone or in combination for 72 h, cells were harvested and re-suspended in an equal volume (ratio 1:1) of PBS and Trypan Blue solution (0.4%) prior to image using EVOS microscope (Life Technology, Carlsbad, CA). Note: The drugs stock solutions were prepared in DMSO. The highest final concentration of DMSO in PBS in the samples was always lower than 0.1%.

To generate the 3D cellular aggregates, cells (1 × 10^4^ cells/well) suspended in medium containing 2.5% (v/v) of Matrigel matrix basement membrane were seeded on ultra-low attachment 96-wells black with clear round bottom plates. After centrifugation (10 min, 1000 rpm), the cells were incubated for 72 h to form spheroids [28]. The spheroids were then treated with various concentrations of drugs for 72 h. The cytotoxicity was evaluated using the CellTiter Glo 3D Luminescent Assay (Promega, Madison, WI), according to the manufacturer’s instructions. The images of the tumor spheroids, before and after drug treatments, were acquired using EVOS FL auto fluorescence microscope (Life Technology, Carlsbad, CA).

### Determination of CI values

Combination Index (CI) values were determined using the widely-used method established by Chou and Talalay [6]. To determine the CI values of a drug combination (drug A + drug B), we first determined the IC_50_ value of each single drug against a specific cell line using a cell viability assay as described above. The cytotoxicity of the drug combination was then evaluated at a specific drug ratio (IC_50_ of drug A: IC_50_ of drug B), using different total drug concentrations. The cytotoxicity study was further extended using multiple drug ratios. The results of the cytotoxicity studies were analyzed using the Compusyn software. The software relied on the median effect equation based on the mathematical model of the physicochemical principal of the mass action law leading to the CI equation:

$$ CI=\sum \limits_{j=1}^n\frac{(D)_j}{{\left({D}_X\right)}_j} $$

where D is the concentration of the drugs in combination to achieve x% of drug effect and D_x_ the concentration of the drugs alone to achieve the same effect. The obtained CI values allow us to assign an antagonistic (CI > 1), an additive (CI = 1), a synergistic (CI < 1), or a strongly synergistic effect (CI < 0.3) of the drug combinations. Based on the cell viability data determined in the human BC cell lines, computer-simulated plots of the CI values of the drug combination (at different drug ratios) versus the cellular fraction affected (Fa = 1 – the ratio of the drug-treated to the non-treated cell numbers) were then generated by the CompuSyn software.

### Selection of drug dosage for cellular studies

For fair comparison, each cell line was treated with the drugs at their corresponding IC_50_ concentrations to induce an equipotency, for insuring each drug contributed to the similar cell killing effect. The BC cells were treated according to the following conditions:

**Table:** 

Breast cancer cell lines	Sal concentration	Das concentration	S + D concentration
MDA-MB-468	0.5 μM	15 μM	15.5 μM
MDA-MB-231	0.1 μM	0.1 μM	0.2 μM
MCF-7	6.5 μM	12 μM	18.5 μM

Note: In the case of MDA-MB-231 cell line, the drugs used at their IC_50_ concentration induced an antagonistic effect **(**Fig. 1d**)**. The concentration of Das was then slightly adjusted (from 0.2 μM to 0.1 μM) to reach the drug ratio 16:15 for achieving a synergistic effect

### Cytotoxicity assays

To investigate the cell death pathway induced by the drug combination, MDA-MB-468 cells (5 × 10^3^ cells/well) were seeded on a 96-well plate overnight, and then treated with various concentrations of S + D (drug ratio fixed at 1:30) in the presence of ferrostatin-1 (Fer-1) and/or necrostatin-1 (Nec-1) for 72 h (1 μM of inhibitor content). The cytotoxicity was then evaluated using the CellTiter Glo Luminescent Assay (Promega, Madison, WI).

To determine whether Sal enhanced the targetability of TNBC by 4-hydroxytamoxifen (Tamo), MDA-MB-468 cells were concurrently treated with Sal and Tamo. Briefly, cells were seeded on 96-well plates (5 × 10^3^ cells/well) in RPMI medium supplemented with 10% charcoal stripped FBS for overnight. The cells were then treated with PBS, Tamo (1 μM), Sal (0.5 μM), or the drug combination for 72 h. The cell viability was measured as described previously. A similar experiment was performed by treating concurrently MDA-MB-468 cells with Das (15 μM) and Tamo (1 μM). For the sequential drug treatment experiments, cells were seeded on T25 culture flasks (1 × 10^6^ cells/flask) overnight and then treated with PBS (control) or Sal (0.5 μM) for 72 h. Cells were then washed twice in PBS, trypsinized, and seeded on 96-wells plates (3 × 10^3^ cells/well) immediately in the presence of various concentrations of Tamo (from 0 to 100 μM) for an additional 72 h period of time, prior to assess the viability.

### Fluorescence-activated cell sorting (FACS)

Intracellular reactive oxygen species (iROS) were measured using a Gallios flow cytometer (Beckman Coulter Inc., Miami, FL). Cells were first seeded on T25 culture flask (1 × 10^6^ cells/flask) overnight and then treated with PBS (control) or the drugs for 6, 12, 24, 48, or 72 h. Drug concentration and ratio were selected according to the IC_50_ values and the synergistic effect as described above. Prior to perform FACS analysis, the cells were incubated with DCF-DA (10 μM) for 30 min, trypsinized, and re-suspended in PBS (1 mL). The analysis was performed on 10,000-gated events (*n* = 3/per sample) at the FL1 channel (λ_ex_ = 488 nm and λ_em_ = 525/20 nm).

Annexin V (AnV)-FITC/propidium iodide (PI) double staining kit was used to evaluate the proportion of apoptotic and necrotic cells. MDA-MB-468 cells, seeded on T25 culture flask (1 × 10^6^ cells/flask), were treated with PBS (control), or the drugs alone or in combination for 72 h. After incubation, the cells were trypsinized and then stained with AnV-FITC (5 μL) and PI (5 μL) for 10 min in the dark prior to FACS analysis. The apoptotic and necrotic cells were detected and quantified using the FL1 and FL2 (λ_ex_ = 488 nm and λ_em_ = 575/20 nm) channels. Healthy, apoptotic, necrotic, and dead cells, were identified as AnV^−^PI^−^, AnV^+^PI^−^, AnV^−^PI^+^, and AnV^+^PI^+^, respectively. The experiment was also performed after treatment of MDA-MB-468 cells with the drug combination in presence of Fer-1 and/or Nec-1 for 72 h (1 μM of inhibitor content).

For determining cell-surface ESR2 level, cells were incubated with drugs alone or in combination for 72 h. The cells were trypsinized and re-suspended in PBS (500 μL), and further incubated with phycoerythrin-labeled anti-ESR2 antibody (1:100 dilution) for 30 min at room temperature. FACS analysis was performed at the FL2 channel.

FACS was also used for cell cycle analysis. After 72 h of incubation with different drug treatments (drug alone or in combination), MDA-MB-468 cells were fixed on ice with a 66% (v/v) ethanol solution in PBS and stored at 4 °C overnight. Cells were then washed twice with PBS and re-suspended in 1X propidium iodide and RNase staining solution (250 μL). Following 30 min of incubation at 37 °C, the cells were analyzed by flow cytometer. The fluorescence of propidium iodide was recorded on the FL2 channel. All the experiments were independently performed in triplicate and the data were processed using Kaluza Software 2.1.1.

### Fluorescence microscopy

MDA-MB-468 or MDA-MB-231 cells (5 × 10^3^ cells/well) were seeded on 8-well Ibidi chamber slides overnight and then treated with PBS (control), Sal (0.5 μM), Das (15 μM), or the drug combination (0.5 + 15 μM) for different time intervals (6, 12, 24, 48, and 72 h). DAPI (9 μM), DCF-DA (10 μM), or phycoerythrin-labeled anti-estrogen receptor β antibody (1:100 dilution) were used for staining the nucleus, the iROS, or the estrogen receptors β (ESR2), respectively, 30 min before imaging. The cells were then washed with PBS. The fluorescence images were acquired using EVOS FL Auto Fluorescence Microscope (Life Technologies, Carlsbad, CA).

### RNA extraction

Cells were seeded on a T25 culture flask (1 × 10^6^ cells/flask) overnight and then treated with PBS (control), drugs alone, or the drug combination for 24, 48, or 72 h. The total RNA was extracted and purified using the RNeasy Mini kit (Qiagen, Hilden, Germany), according to the manufacturer’s instructions. The final RNAs was quality-controlled using Agilent 2100 Bioanalyzer and quantified by absorbance using NanoDrop, prior to be analyzed by RNA-seq or Reverse Transcription-quantitative Polymerase Chain Reaction (RT-qPCR).

### Transcriptome analysis

Library was constructed on the purified RNAs obtained from the PBS- or drug-treated MDA-MB-468 cells (4 biological replicates per condition), using Illumina TruSeq RNA preparation kit (Illumina, San Diego, CA). The samples were sequenced using HiSeq4000 sequencer with single-end 50 bps reads. The raw sequencing reads in BCL format were processed through bcl2fastq 2.19 (Illumina) for FASTQ conversion and demultiplexing. The RNA reads were aligned and mapped to the GRCh37 human reference genome by STAR (Version2.5.2) [29]. The transcriptome reconstruction was performed by Cufflinks (Version 2.1.1). The abundance of transcripts was measured with Cufflinks in Fragments Per Kilobase of exon model per Million mapped reads (FPKM) [30]. Gene expression profiles were constructed using the DESeq2 package [31]. The resulting corrected *p*-values were calculated based on the Benjamin-Hochberg’s method to adjust multiple testing and control the false discovery rate. Finally, Ingenuity Pathway Analysis (IPA 4.0, Ingenuity System) was used to model the differential gene expression data. The following cutoffs: adjusted *p*-value (*P*_adj_) < 0.01 and log2 fold gene expression change > 1.5 were applied prior to performing the data analysis. The raw RNA sequencing data reported in this paper is available in the Gene Expression Omnibus (GEO) database using the accession number GSE135514 and following the link:

https://www.ncbi.nlm.nih.gov/geo/query/acc.cgi?acc=GSE135514.

### Reverse transcription-quantitative polymerase chain reaction (RT-qPCR)

The first-strand cDNA library was synthesized from RNA samples (2 μg) using M-MLV reverse transcriptase reagent kit (Promega, Madison, WI). Quantitative real-time PCR was performed after adding the cDNA products (1 μL), the corresponding gene primer set (20 μM; 1 μL), and SYBR Green Master Mix (10 μL) to ultrapure water (13 μL). Forty-five cycles of qPCR gene amplification were performed. StepOnePlus Real-Time PCR system (Applied Biosystems, Foster City, CA) was used to conform the extension step. The number of cycles (C_T_) were normalized and corrected from the house keeping gene glyceraldehyde 3-phosphate dehydrogenase (GAPDH). Differential gene expression was expressed as a relative fold change compared to the results of the control cells treated with PBS only, using the comparative C_T_ method [32]. The sequences of the primers, listed in **Fig.****S1**, were designed using the National Center for Biotechnology Information (NCBI) software.

### Western blot

MDA-MB-468 or MDA-MB-231 cells were seeded on T25 culture flask (1 × 10^6^ cells/flask) overnight and treated with PBS (control), Sal (0.5 μM), Das (15 μM), or the drug combination for 72 h. Cells were then collected and lysed using RIPA buffer supplemented with 1% of phenylmethylsulfonyl fluoride (200 mM), 1% of protease inhibitor cocktail, and 1% of sodium orthovanadate (100 mM) (Santa Cruz Biotechnology Inc., Dallas, TX). The total protein contents in cell lysates were quantified using a microBCA assay (ThermoFisher Scientific, Waltham, MA). The samples (15 μg of proteins) were separated by NuPAGE™ 4–12% Bis-Tris Gel at 120 V and were subsequently transferred onto a polyvinylidene difluoride membrane. The membranes were blocked with 1X Tris buffered saline containing 0.1% of Tween (v/v) (TBST) and 8% (w/v) of skimmed milk for 1 h at room temperature, and then incubated with primary antibodies overnight at 4 °C. Membranes were washed 3 times with TBST buffer for 10 min and incubated with a 1:5000 dilution of the peroxidase-conjugated secondary antibody for 1 h at room temperature. Membranes were finally washed 3 times with TBST buffer for 10 min. The bound secondary antibodies were detected using SuperSignal West Pico PLUS Chemiluminescent Substrate. The chemiluminescent signals were collected using the Odyssey Two-color Infrared Laser Imaging System (Li-cor, Lincoln, NE) and the blots were processed and cropped using Image Studio Lite 5.2 software.

### siRNA transfection

To knockdown the ESR2 expression induced by the Sal treatment, MDA-MB-468 cells (5 × 10^4^ cells/well) were seeded on a 6-well plate overnight and then simultaneously treated with Sal (0.5 μM) and transfected with Silencer Select siRNA oligonucleotides (ThermoFisher Scientific, Waltham, MA), according to the manufacturer instructions. Briefly, siRNA (60 nM) was mixed with RNAiMAX transfection reagent in the presence of OptiMEM reduced serum medium for 5 min at room temperature. The siRNA-lipid complex and Sal were then co-incubated with the cells. An equimolar mixture of 3 different pre-designed siRNA (20 nM each) targeting multiple regions of ESR2 gene **(Table****S1****)** was used to silence the estrogen expression. Silencer control 1 (ctl1) and control 2 (ctl2) siRNA, not able to interact with any human RNA, were used as negative controls. 72 h after siRNA transfection and drug treatment, cells were harvested prior to performing FACS analysis and cell viability assay.

## Results

### Sal and Das synergistically inhibited different BC cell lines and spheroids

To investigate the cytotoxicity of our drug combination (S + D), we first determined the inhibitory concentration (IC_50_) values of Sal or Das on different human BC cell lines: MDA-MB-468, MDA-MB-231, and MCF-7. According to the specific IC_50_ values obtained, we applied different drug ratios and concentrations of the drug combination and evaluated the cytotoxic effects. Based on the cell growth inhibition, we calculated the combination index (CI) value of each dosage regimen using Compusyn software, and ultimately determined whether the drug combination was synergistic (CI < 1), additive (CI = 1), or antagonistic (CI > 1) [33]. Our results showed that Sal was generally more potent than Das regardless of the cell lines tested (Fig. 1b). Among the tested cell lines, the drug was more cytotoxic against MDA-MB-468 and MDA-MB-231 than MCF-7 (Fig. 1c). On the other hand, Das displayed relatively higher, micromolar range IC_50_ values on MDA-MB-468 and MCF-7 than MDA-MB-231. The results were expected since both MDA-MB-468 and MCF-7 are intrinsically resistant to Das [34]. We noted that for most drug combinations, the degree of synergism varied with the applied drug ratio and the tested cell lines (**Fig.****S2**) [35–37]. Similarly, we recognized that our drug combination offered a stronger (with lower CI values) and more reliable synergistic effect on MDA-MB-468 than MCF-7. We were able to use a variety of drug ratios and concentrations against MDA-MB-468 while maintaining synergism (**Fig.****S3****A**). On the other hand, although the drug combination could synergistically inhibit MCF-7, applying certain drug ratios, such as 1:60 (Sal:Das) for treatment resulted in an undesired antagonistic effect (Fig. 1d). Interestingly, both MDA-MB-468 and MDA-MB-231 are triple negative BC (TNBC) cells but they responded differently to the treatment. The drug combination primarily offered an antagonistic rather than a synergistic effect with regard to MDA-MB-231 (Fig. 1d). Nevertheless, we could formulate a couple of drug ratios (16:15 and 32:15) that endorsed synergism to the 3 tested cell lines. The synergistic effect of the drug combination in each cell line was further confirmed with Trypan Blue exclusion assay **(Fig.****S4****).**

Tumor spheroids offer more accurate tumor-mimicking models than the traditional cell culture systems [38]. For this reason, we also evaluated the cytotoxic effects of Sal and Das using tumor spheroids. We first prepared MDA-MB-468, MDA-MB-231, and MCF-7 spheroids in 96-well round-bottom plates (Figs. 1e and f). We then determined the combined cytotoxic effects of Sal and Das using the same approach we applied to our cell culture studies. In general, Sal was more potent than Das regardless of the tested spheroids. Compared to the monolayer cell culture method, we needed to apply a higher drug concentration in order to effectively eradicate the tumor spheroids, as indicated by the relatively higher IC_50_ values of Sal and Das (Figs. 1g and h). The spheroids were significantly more resistant to the drug treatments probably due to the complexity of the 3D structure as well as the limited penetration and diffusion of the drugs into the aggregates [39]. In the cell culture studies, the drug combination showed synergism against MDA-MB-468 and MCF-7 (**Fig.****S3****B**). However, there were remarkable differences in terms of the optimal drug ratio used for treating cell lines and tumor spheroids (Figs. 1h and i). A drug ratio of 32:15 had a strong synergistic effect on MDA-MB-468 cells, but an antagonistic effect on the spheroids. In fact, the optimal drug ratio offering the strongest synergistic effect on the spheroids was 1:30. Further studies are required to investigate whether the observed discrepancies originated from differences in spheroidal penetration, and thus cellular uptakes, of the 2 drugs [40, 41]. Overall, we demonstrated that our drug combination synergism was volatile, and strongly relied on the applied drug ratio as well as the employed cell culture and tumor spheroid models. Nevertheless, for the three BC cell lines tested, the drug combination was more effective to fragment the 3D spheroids compared to the drugs alone, as shown by the increase of the spheroids size **(Fig.****S5****).** Despite the fact that it was feasible to obtain synergism for MDA-MB-468, MDA-MB-231, and MCF-7 by fine-tuning the drug ratio, for ease of comparison, we chose cell cultures for further studying the synergistic mechanism of the drug combination at the molecular level.

### Sal and Das induced intracellular ROS (iROS) in BC cell lines in a time-dependent manner

**Fig. 2:**
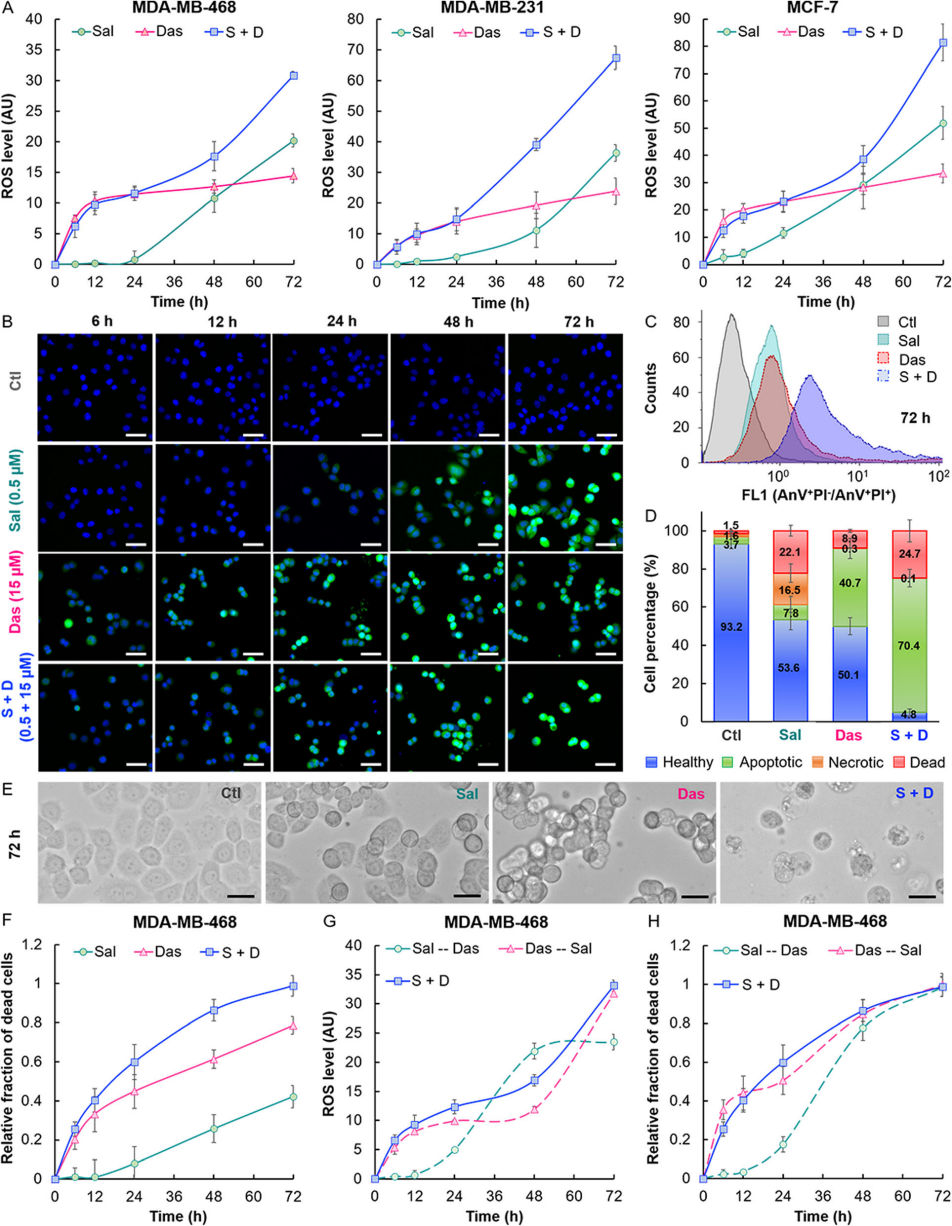
The drug combination enriched iROS production and promoted cytotoxicity compared to Sal or Das alone, in a time-dependent manner. **a** Plots of the drug-induced iROS level versus time. The induction of iROS by the drug combination followed a 2-step kinetic. MDA-MB-468, MDA-MB-231, and MCF-7 cell lines were treated with individual drugs (at the corresponding IC_50_ concentration (Fig. 1c)) or the 2-drug mixture prior to incubation with DCF-DA for FACS analysis of the iROS level. The mean fluorescence was calculated by comparison with PBS-treated cells (control). **b** Fluorescence microscopy confirmed that the drug-induced iROS increase in treated MDA-MB-468 cells was time-dependent. Prior to imaging, the cells were treated with DCF-DA and DAPI for staining the iROS (green) and nucleus (blue), respectively. Scale bar is 45 μm. **c** A flow cytometry graph showing the increase of the apoptotic (AnV^+^PI^−^)/dead (AnV^+^PI^+^) cell population in response to the drug treatments. MDA-MB-468 cells were incubated with Sal (0.5 μM), Das (15 μM) or the 2-drugs combination for 72 h prior to staining with AnV-FITC and PI for FACS analysis. **d** Graph bars showing the percentage of healthy, apoptotic, necrotic, and dead cells following treatment with PBS (control) Sal, Das, or S + D for 72 h (see **Fig.****S6** for detailed quantification of the cell populations). **e** Representative microscopic images of MDA-MB-468 cells 72 h after drug incubation. Scale bar is 25 μm. **f** Cell viability assay showed that the cytotoxicities of Sal and Das alone or the drug combination were also time-dependent. **g-h** Comparing the cytotoxicities of the same drug combination applied sequentially and concurrently for treating MDA-MB-468 cell line. The cells were treated sequentially with Sal and followed by Das (Sal--Das) or Das and then Sal (Das--Sal), or concurrently with Sal and Das (S + D). The concentration of Sal and Das used in this study was 0.5 and 15 μM, respectively. Plots showing the changes in the (**g**) iROS level and (**h**) ratio of the drug-treated to non-treated cell numbers over time. All the experiments were independently performed in triplicate

Sal has been shown to induce iROS production, suppress the phosphoinositide 3-kinase/protein kinase B/mammalian target of rapamycin signaling pathways (PI3K/AKT/mTOR), and cause apoptosis in prostate, brain, and breast cancer cells [42–44]. Das can also induce iROS [45]. These provided the rationale for us to investigate whether the drug combination would enhance the ROS increase in different human BC cell lines including MDA-MB-468, MDA-MB-231, and MCF-7, and thereby promote cytotoxicity compared to using Sal or Das alone. To test this hypothesis, we measured the kinetic changes of iROS levels in cells treated with Sal and/or Das using flow cytometry. Subsequent fluorescence-activated cell sorting (FACS) analysis revealed that there was a lag of iROS induction by Sal (Fig. 2a). The drug only began to induce iROS 12 to 24 h after incubation (depending on the tested cell lines). In contrast, Das rapidly induced iROS, with the level reaching a plateau after 24 h. Despite the iROS increase after an initial transient phase, Sal was able to induce more iROS over a longer period of time (72 h) compared to Das (24 h). When the cells were concurrently treated with Sal and Das, the increase in the iROS level followed a 2-step kinetic. Presumably the 2 drugs worked together to serially induce ROS, first by Das and then by Sal. We further examined the drug-induced iROS in MDA-MB-468 cells under a fluorescence microscope, using dichlorofluorescein diacetate (DCF-DA) as the staining for visualization. Green fluorescence (iROS) began to appear in cells 24 h after Sal incubation (Fig. 2b). Nearly all cells treated with Das showed fluorescence signals as early as 6 h. Compared to each drug alone, the drug combination induced more apoptosis, as shown by an increase of the apoptotic (AnV^+^PI^−^) and (AnV^+^PI^+^) cell populations accompanied with a decrease of the number of healthy cells **(**Figs. 2c-d **and****S6****).** AnV is known for detecting cell-surface exposure of phosphatidylserine triggered by apoptosis, ferropotosis, and/or necroptosis. Here, an addition of ferrostatin-1 (Fer-1) and/or necrostatin-1 (Nec-1) did not rescue the cell death, suggesting the cell-killing effect was unlikely from the results of ferropotosis or necroptosis **(Fig.****S7****).** Phase-contrast microscopic imaging also showed that cells treated with the drugs became unhealthy (shrank) over time (Fig. 2e). This prompted us to further investigate whether the cytotoxic effects of Sal and Das were also time-dependent. As expected, Sal showed a lag of the cytotoxic effect on MDA-MB-468 cells. A plot of the ratio of non-treated cell number to treated cell number with time revealed a 12 h delay of the cytotoxic effect (Fig. 2f). In contrast, Das rapidly executed its desired drug activity. Interestingly, we were able to obtain a similar iROS generation and cytotoxic profiles whether the drugs were given concurrently or sequentially with Das and then followed by Sal (Figs. 2g and h). However, reversing the order of the drug incubations (Sal followed by Das) significantly weakened the iROS production as well as the cytotoxic effect during the first 24 h of treatment (Figs. 2g and h).

### Sal and Das suppressed genes regulated by STAT3, Wnt/β-catenin, and Hedgehog cell signaling pathways

**Fig. 3:**
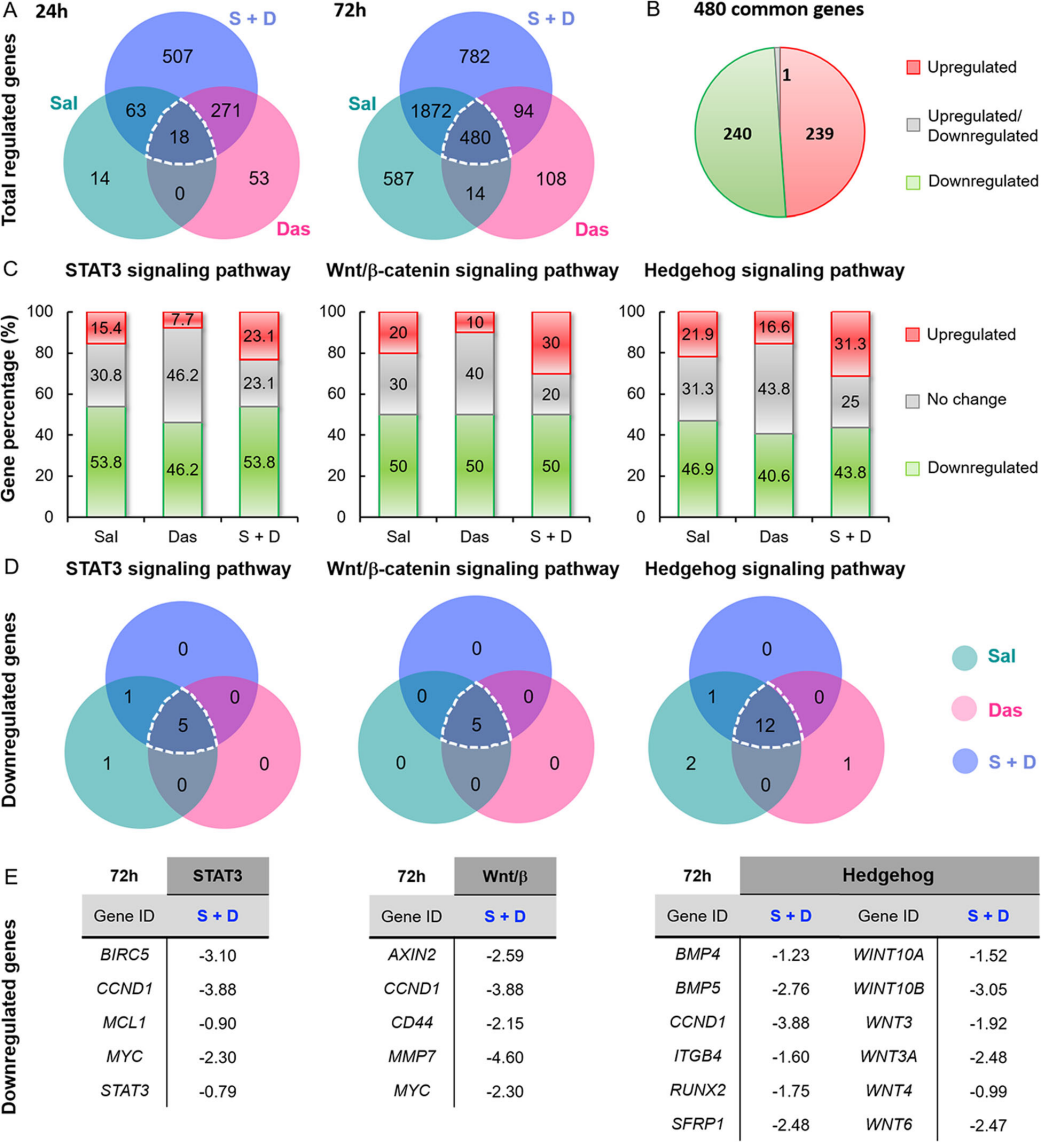
Inhibition of STAT3, Wnt/β-catenin, and hedgehog signaling pathways by Sal and Das. **a** Venn diagrams showing the total number of genes that were commonly regulated (downregulated and upregulated) by the 3 different drug treatments (Sal, Das, and S + D). MDA-MB-468 cells were incubated with the drugs for 24 h and 72 h prior RNA-seq analysis. **b** A pie chart showing the number of genes that were commonly upregulated and downregulated among the treatment conditions. **c** Graph bars showing the percentage of drug-induced upregulation and downregulation of the targeted genes that are known to be modulated by the STAT3 (13 genes), Wnt/β-catenin (10 genes), and hedgehog (32 genes) pathways (also see **Fig.****S8** for the full list of the genes analyzed). MDA-MB-468 cells were treated with drugs alone or in combination for 72 h prior to extract the mRNAs for RNA-seq analysis. The experiments were independently performed in quadruplicate. **d** Venn diagrams showing a significant overlapping of the activities between Sal and Das. **e** Lists of the genes that were commonly downregulated by Sal, Das, and the drug combination

The mechanisms of action of both Sal and Das are complex. It has been widely reported that these two drugs regulate multiple signaling pathways including STAT3, Wnt/β-catenin, and hedgehog [16–18, 46, 47]. RNA-seq is a combinatorial technique that allows for quantifying global gene expression in biological samples. We employed this next generation sequencing technique to provide an initial insight into how Sal and Das alone, as well as in combination, modulated gene expression in the MDA-MB-468 cell line at 24 and 72 h. Each drug treatment displayed a unique gene expression profile**.** We found that the number of genes that were modulated, whether they were upregulated or downregulated, regardless of the treatments, increased over time (**Table****S2**). Genes that were commonly regulated by the drugs alone or the drug combination increased from 18 to 480 over time **(**Fig. 3a**).** Among all the common 480 genes, 239 and 240 of them were upregulated and downregulated, respectively. (Fig. 3b). We then investigated the expression of the downstream targeted genes that are known to be regulated by Sal via modulations of the STAT3 (13 genes), Wnt/β-catenin (10 genes), and hedgehog (32 genes) pathways (**Fig.****S8**). Among all these genes that we analyzed, *CCND1* (which encodes cyclin D1) was the only one that has been reported to be regulated by all 3 pathways. *MYC* (which encodes myc) is the common targeted gene of the STAT3 and Wnt/β-catenin pathways. According to the differential expression of the genes, we found that more than 40% of the genes associated with the 3 pathways were suppressed by either Sal or Das (Fig. 3b). As expected, most of the genes that were downregulated by Sal were also downregulated by Das (Fig. 3c). However, the drug combination did not seem to increase the number of the genes being modulated. Further, only 10 out of the 50 genes that we analyzed were either additively or synergistically suppressed by the drug combination (**Fig.****S8**). Despite a predicted significant overlap in the activities of Sal and Das (Fig. 3d-e), using the drug combination only partially enhanced the suppression of certain downstream targeted genes known to be regulated by the STAT3, Wnt/β-catenin, and hedgehog pathways. Those results strongly suggested that Sal and Das might display their synergistic effect through alternative cell signaling pathways.

### Sal and Das exhibited synergistic effect of cell cycle arrest through a partial suppression of the estrogen-mediated S-phase entry pathway

**Fig. 4:**
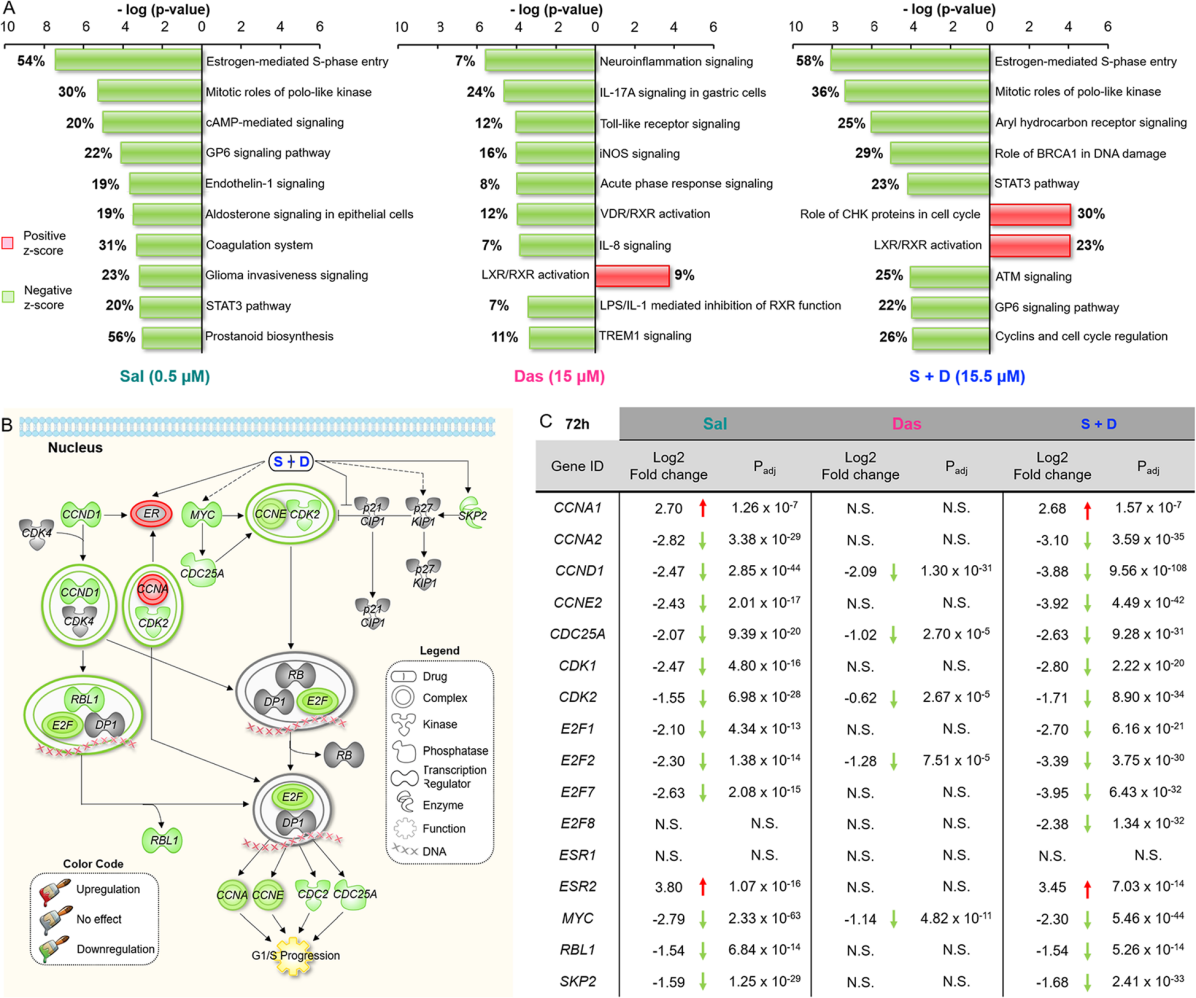
Sal and Das modulated multiple cellular pathways. **a** Bar graphs showing the 10 most significant canonical pathways that were modulated by the drugs alone or in combination. The estrogen-mediated S-phase entry pathway was found to be the most significant one suppressed by the drug combination. The number next to each bar represents the percentage of modulated genes associated with the pathway. MDA-MB-468 cells were treated with Sal (0.5 μM) or Das (15 μM) or the drug combination for 72 h prior to perform the RNA-seq analysis. **b** A diagram showing how the genes associated with the estrogen-mediated S-phase entry pathway was significantly suppressed by the drug combination. **c** A table summarizing the differential gene expression of cells treated with drugs alone or together. The differential gene expression was presented as a log2 fold change relative to the corresponding gene expression in cells treated with PBS (control). The experiments were performed in quadruplicate. N.S. = no significant change of the gene expression

To investigate the synergistic mechanisms of the drug combinations, we used Ingenuity Pathway Analysis software (IPA 4.0) to identify any significant canonical pathways that were modulated by the drug treatments, based on global differential gene expression in MDA-MB-468 cell lines. Our results showed that the estrogen-mediated S-phase entry pathway was the most significant one modulated by Sal after 72 h of treatment (Fig. 4a). The pathway is composed of 26 genes/proteins working together as gatekeepers for G1/S phase progression (Fig. 4b). Sal modulated 54% of the genes (14 out of 26 genes), with 12 of them downregulated. The Sal-induced gene expression changes were time-dependent, as there was a limited transcriptomic change when the cells were treated with Sal for only 24 h (**Fig.****S9**). On the other hand, Das could only suppress 5 of the genes associated with the estrogen-mediated S-phase entry pathway, which included *CCND1*, *CDC25A*, *CDK2*, *E2F2*, and *MYC* (Fig. 4c). *CCND1* and *MYC* are the downstream targeted genes of the STAT3, Wnt/β-catenin, and/or hedgehog pathways (see the above section). We also discovered that either Sal or Das was able to suppress *E2F2*. Most importantly, following the 2-drugs combination treatment, the proportion of genes modulated in the estrogen-mediated S-phase entry pathway increased from 54 to 58% compared to Sal monotherapy **(**Fig. 4a**)**. This strongly suggests that the modulation of the estrogen-mediated S-phase entry pathway is mainly induced by Sal but the addition of Das further contributed to its inhibition, as Das enhanced the suppressions of most genes (10 out of 12) found downregulated by Sal (Fig. 4c). Interestingly, although Sal suppressed 54% of the genes associated with the estrogen-mediated S-phase entry pathway, it upregulated estrogen receptor (ER). It is noted that there are two classes of ER: ERα and ERβ. The tumors of ER-positive breast cancer patients are overexpressed with ERα. Here, Sal, Das, or the drug combination did not alter the ERα expression in MDA-MB-468. In fact, ERβ was found to be upregulated by Sal, but not Das. Further study is needed to investigate how Sal regulates ERβ.

**Fig. 5:**
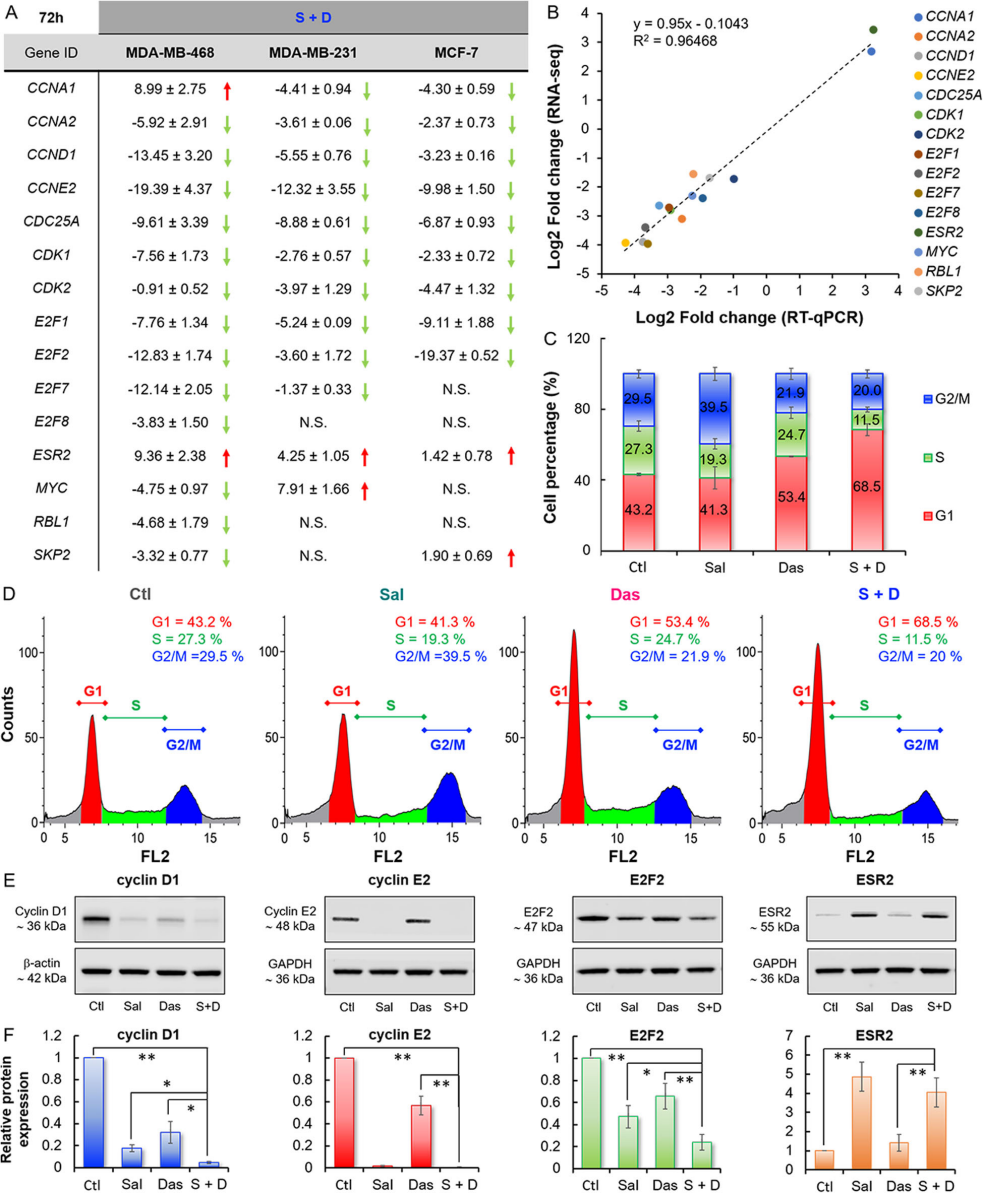
The drug combination enhanced cell cycle arrest at the G1/S phase. **a** A table summarizing the changes in the expression of genes (induced by the drug combination) associated with the estrogen-mediated S-phase entry pathway. The mRNAs were extracted from different BC cell lines 72 h after treatment with the drug combination prior to RT-qPCR analysis. **b** A correlation plot of the differential gene expression levels in MDA-MB-468 cell lines determined using RNA-seq and RT-qPCR methods. **c** Plots of cell counts versus the propidium iodide fluorescence (FL2). MDA-MB-468 cells were treated with PBS (control), Sal (0.5 μM), Das (15 μM), or the drug combination for 72 h, fixed in ethanol, and incubated with propidium iodide and RNase staining solution prior to FACS analysis. All the experiments were performed in triplicate. **d** Graph bars showing the percentage of cells at G1, S, and G2/M phases after the drug treatments. **e** The drug combination also modulated the estrogen-mediated S-phase entry pathway at the translational level. Western Blot analysis of cyclin D1, cyclin E2, E2F2, and ESR2 protein expression in MDA-MB-468 cells after exposure to the drugs alone or in combination for 72 h. GAPDH and β-actin were used as loading controls based on the molecular weight of the proteins of interest. The blots were processed and cropped using Image Studio Lite 5.2 software. Full-length blots are available **Fig.****S13**. **f** Graph bar showing the expression level of each selected protein. The relative protein expression levels were quantified using Image Studio software and normalized to the control cells treated with PBS. All the experiments were performed in triplicate. Data were presented as mean ± standard deviation (SD) and statistical differences were analyzed using Student’s *t*-test (*P < 0.05, **P < 0.01)

We further validated the RNA-seq data using RT-qPCR analysis of the gene expressions that are associated with the estrogen-mediated S-phase entry pathway (Figs. 5a **and****S10****).** There was a linear relationship (R^2^ > 0.96) between the 2 methods used for determining differential gene expression induced by the drug combination (Figs. 5b **and****S11**). Using RT-qPCR, we also demonstrated that the drug combination synergistically suppressed the estrogen-mediated S-phase entry pathway in MDA-MB-231 and MCF-7, in addition to MDA-MB-468 cell lines (Figs. 5a **and****S12**). As mentioned above, this pathway controls the transition from G1 to S phase in cell cycle. An inhibition of the pathway can induce cell cycle arrest [48]. To confirm this effect, MDA-MB-468 cells were treated with the drugs alone or in combination for 72 h and were then analyzed by flow cytometry. As expected, Sal decreased the percentage of cell population in the S phase from 27.3% (non-treated control) to 19.3% (Figs. 5c and d). The drug combination further enhanced such a decrease to 11.5%, which accompanied an increase of the cell population at the G1 phase from 43.2% (control) to 68.5%. Overall, our results suggested that the synergistic effect of the drug combination was possibly achieved through promotion of the cell cycle arrest via partial inhibition of the estrogen-mediated S-phase entry pathway. This was further supported by western blot analysis of the translational products (protein expression). Either Sal or Das downregulated cyclin D1 (CCND1), cyclin E2 (CCNE2), and E2F2 (Figs. 5e-f **and****S13**). Importantly, the drug combination enhanced the suppressions of cyclin D1 and E2F2.

### The therapeutic implication of Sal-induced *ESR2* expression

**Fig. 6:**
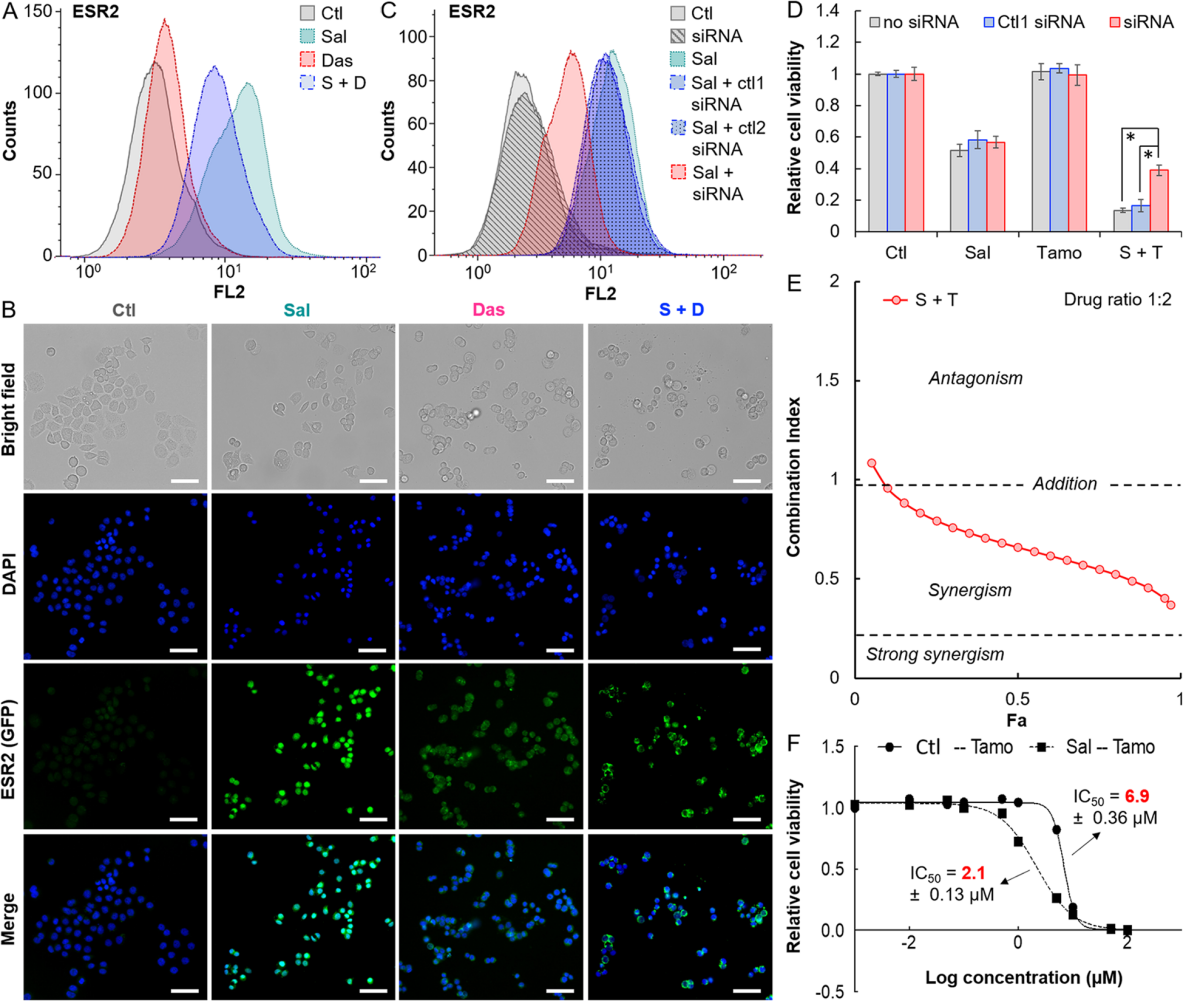
Sal-induced the targeting of TNBC. **a** Sal induced ESR2 in MDA-MB-468 cell line. A flow cytometry graph showing an upregulation of ESR2 expression in response to Sal or the drug combination of Sal and Das treatment. The cells were treated with drugs alone (at the corresponding IC_50_ concentration **(**Fig. 1c**)**) or 2-drugs combination prior to incubation with phycoerythrin-labeled anti-ESR2 for FACS analysis. **b** Representative fluorescence microscopic images showing Sal induced ESR2 in MDA-MB-468 cells. Prior to imaging, the cells were incubated with phycoerythrin-labeled anti-ESR2 and DAPI for staining the ESR2 receptors (green) and the nucleus (blue), respectively. Scale bar is 60 μm. **c** Flow cytometry graph showing that the ESR2 expression induced by the salinomycin treatment was knocked down with siRNA. MDA-MB-468 cells were simultaneously treated with Sal (0.5 μM) and transfected with Silencer Select ESR2 siRNA oligonucleotides for 72 h. Ctl1 and ctl2 siRNA were used as negative controls. **d** Plots of the relative cell viability of MDA-MB-468 cells 72 h after treatment with Sal, Tamo, or the drug combination (S + T) with no siRNA transfection or in the presence of ctl1 siRNA (negative control) or with siRNA of ESR2. Data were presented as mean ± standard deviation (SD) and statistical differences were analyzed using Student’s *t*-test (*P < 0.05). **e** Synergistic cytotoxicity of Sal and Tamo. Stimulated plots of the CI values of the drug combination at a drug ratio of 1:2 versus the cellular fraction affected (Fa) values. The plots were generated using the CompuSyn software. All the experiments were independently performed in triplicate. **f** Comparing the cytotoxic effect of Tamo on MDA-MB-468 cells pre-treated with Sal and PBS. The cells were pre-treated with PBS (control) or Sal (0.5 μM) for 72 h and then be trypsinized. Same number of cells were re-seeded and immediately treated with Tamo (1 μM) for 72 h. The results were fit into sigmoidal dose response curves for calculating the IC_50_ values. The experiments were independently performed in triplicate

Although the IPA software identified the estrogen-mediated S-phase entry pathway as the main pathway modulated by the drug combination, Sal surprisingly induced rather than suppressed *ESR2* expression (Figs. 4b and c). Western blot analysis showed an increase in the translational product, estrogen receptor β (ESR2), in MDA-MB-468 treated with Sal (Figs. 5e and f**)**. We further confirmed the such a Sal-induced ESR2 with FACS analysis and microscopic study. Compared to the non-treated cells, MDA-MB-468 treated with Sal showed an approximately 10-fold increase of the fluorescence signal with fluorophore-conjugated ESR2 antibody staining (Figs. 6a and b). The drug-induced ESR2 could be knocked down by siRNA **(**Fig. 6c**).** Similarly, we also observed an increase of ESR2 expression in MDA-MB-231 cells treated with Sal (**Fig.****S14**).

Tamoxifen has been shown to increase the interaction between ESR2 and mutant TP53, and leading to a reactivation of TP73 and apoptosis [49]. The ability of Sal to induce ESR2 in MDA-MB-468 and MDA-MB-231 suggested that there is potential of a new drug-induced targeting of ESR2 approach for BC. 4-hydroxytamoxifen (Tamo) is an active metabolite of tamoxifen known to strongly bind estrogen receptors (**Fig.****S15****A**). Here, we applied 1 μM of Tamo for investigating whether the drug could effectively target Sal-induced TNBC (Fig. 6d**)**. At this drug concentration, Tamo was non-toxic to MDA-MB-468 since the cells only displayed a very low level of ESR2 (Fig. 5e). Using Sal (0.5 μM) and Tamo concurrently reduced the cell viability to 10% compared to the cells treated with PBS (100%). The drug combination was more cytotoxic than the single drug treatment, as Sal alone could only reduce the cell viability to 50%**.** On the other hand, Das did not induce ESR2 expression and thus its cytotoxicity was not being enhanced by Tamo (**Fig. S****15****B**). A plot of the CI values of the drug combination versus cellular fraction affected (Fa) revealed a strongly synergistic effect of the two drugs (Fig. 6e). Such an enhanced cytotoxicity was attributed to the induction of ESR2 by Sal, as Tamo could no longer enhance the toxicity of Sal when the ESR2 expression was knocked down by siRNA (Fig. 6d). To demonstrate that Sal-induced ESR2 sensitized the targetability of TNBC cells by Tamo, we first pre-treated MDA-MB-468 with Sal and followed by Tamo treatment. Cytotoxicity assay revealed that Tamo was more effective on cells pretreated with Sal than PBS, with IC_50_ values of 2.1 versus 6.9 μM (Fig. 6f). Overall, our results showed that Sal-induced ESR2 expression worked complimentarily with Tamo to promote an anticancer effect, suggesting a novel targeted treatment approach for BC lacking of clinically-relevant biomarker, such as TNBC.

## Discussion

A recent study supported the benefits of targeting Src-STAT3, which showed that Das and CYT387 (JAK/STAT inhibitor) together synergistically reduced proliferation and induced apoptosis of renal cell carcinoma [50]. In the present study, we demonstrated that Sal and Das (Src inhibitor) together synergistically inhibited the growth of MCF-7 and MDA-MB-468 cell lines. However, similar to other reported drug combinations [35–37], the degree of synergism relied on the applied drug ratio, and was also cell line-dependent. ROS are central signaling molecules that regulate cell growth and survival. We showed that Das rapidly elevated the iROS level inside BC cells. In contrast, cells treated with Sal followed an initial transient phase- a lag of iROS generation, depending on the treated cell types. The delayed iROS production held back the cytotoxic effect of Sal. This was supported by a previous report that Sal-induced iROS act upstream of the PI3K/AKT/mTOR signaling pathway [44]. The drug was shown to decrease phosphorylation of AKT and mTOR, and led to apoptosis in a time- and concentration-dependent manner. The difference in the kinetics of drug-induced iROS between Sal and Das resulted in a 2-step kinetic profile when they were concurrently used in a combination setting. Nevertheless, the drug combination generated more iROS and displayed enhanced cytotoxicity compared to Sal or Das alone.

In terms of drug mechanism, Sal inhibits STAT3, Wnt/β-catenin, and hedgehog cell signaling pathways [16–18, 46, 47]. These pathways are composed of multiple protein kinases, leading to overall activities that are often controlled by a balance (ratio) between the phosphorylated and non-phosphorylated forms of the enzymes rather than the actual expression levels. Therefore, we applied an indirect approach to investigate how Sal and Das regulated the activities of these pathways: we simultaneously determined the differential expression of the downstream targeted genes using RNA-seq. The Sal-induced inhibitory effect resulted in many of the targeted genes involved in the 3 pathways being downregulated. These genes were also found suppressed by Das [50–52]. Despite the fact that we observed an overlapping of the drug-induced transcriptomic changes between Sal and Das, the drug combination did not offer a superior advantage in terms of enhancing gene regulations, except in the cases of certain genes such as *BIRC5*, *CCND1*, and *Wnt3A*, which were synergistically suppressed. This suggested that the 3 pathways might only partially contributed to the enhanced cytotoxic effect. In fact, IPA software showed that Sal significantly suppressed the estrogen-mediated S-phase entry pathway. This pathway controls G1/S phase transition. Sal induces cell cycle arrest [53]. Fourteen out of 26 (54%) of the genes associated with the pathway were modulated by the drug, with 12 of them being downregulated. Among them, *CCND1* is a common downstream transcriptional target of the STAT3, Wnt/β-catenin, and hedgehog pathways. A suppression of *SKP2* (encoding Skp2) was expected here given that Sal is known to inhibit the transcription of *SKP2* [13]. For the first time, we discovered that Sal also downregulated *E2F1*, *E2F2*, and *E2F7*. These genes belong to a family of transcription factors, E2F, that is functionally divided into activators (E2F1-E2F3) and inhibitors (E2F4-E2F8) [54, 55]. The exact roles of the individual E2F homologues in cancer progression are not fully understood. E2F1-E2F3 are the central regulators of cell cycle [56]. The activities are negatively regulated via binding with retinoblastoma-like 1 protein (RB; encoded by RBL1). During the late G1 phase of the cell cycle, cyclin-dependent kinase complexes (cyclin D/CDK4) phosphorylate RB to release the active E2F. This promotes the transcriptions of multiple targeted genes required for proceeding to the S-phase, including those that were downregulated by Sal (*CCNA2*, *CCNE2*, *CDC2*, and *CDC25A*) in the present study. Das induces apoptosis and cell cycle arrest via modulations of EGFR/MAPK/AKT signaling pathways [23]. This might explain why the drug could also suppress *CCND1*, *CDC25A*, *CDK2*, *E2F2*, and *MYC* here. When used with Sal, Das enhanced the inhibition of the estrogen-mediated S-phase entry pathway. This was confirmed by RT-qPCR for mRNA expression and western blot for the protein levels. Using RT-qPCR, we further showed that the drug combination also effectively downregulated the pathway in MCF-7 and MDA-MB-231 cell lines. An inhibition of the estrogen-mediated S-phase entry pathway by the drug combination led to a more severe cell cycle arrest compared to Sal or Das, as shown in our cell-cycle assays.

ESR2 was only discovered recently, and the exact role of ESR2 remains elusive. The receptor is evenly distributed among the four molecular BC subtypes, including TNBC [57, 58]. Early studies showed that ESR2 exhibited anti-tumor properties. Introducing exogenous ESR2 cDNA to MDA-MB-231 inhibited the cell proliferation [59]. Following to treatments with ESR2 agonists (ERB-041 and WAY200070), both MDA-MB-231 and HS578T decreased the invasiveness [60]. In another study, ESR2 was shown to bind and oppose the transcriptional activity of mutant PT53, and led to an inhibition of epithelial-to-mesenchymal transition in MDA-MB-231 [61]. On the other hand, many studies conversely reported that ESR2 was in fact pro-tumorigenic. Using shRNA to silence ESR2 reduced TNBC cell proliferation [62]. An activation of ESR2 with diarylpropionitrile, a specific agonist, increased the proliferation and migration of TNBC cells [57]. Clinically, the role of ESR2 is also controversial. The presence of ESR2 in BC tumor was associated with the levels of proliferation markers such as Ki67 [63], but a high expression of the receptor was shown to improve the overall survival of BC patients treated with tamoxifen [64, 65]. In TNBC, ESR2 expression was found correlated with a lower overall relapse-free survival [57]. It has now become more clear that ESR2 has a bi-faceted role. Whether ESR2 displayed a pro- or anti-proliferative effect depended on the binding to the wild type or the mutated form of P53, respectively [49]. The same study also revealed that Tamo, a competitive ESR receptor, increased the interaction between ESR2 and mutant TP53 (found in MDA-MB-468 and MDA-MB-231 cell lines), and leading to a reactivation of TP73 and apoptosis [49]. In the present study, we discovered a novel mechanism of Sal to induce ESR2 expression in TNBC cell lines. Using a drug combination of Sal and Tamo could produce a synergistic anti-cancer effect against both MDA-MB-468 and MDA-MB-231 cell lines, suggesting a novel drug-induced targeting approach for TNBC treatment.

**Fig. 7:**
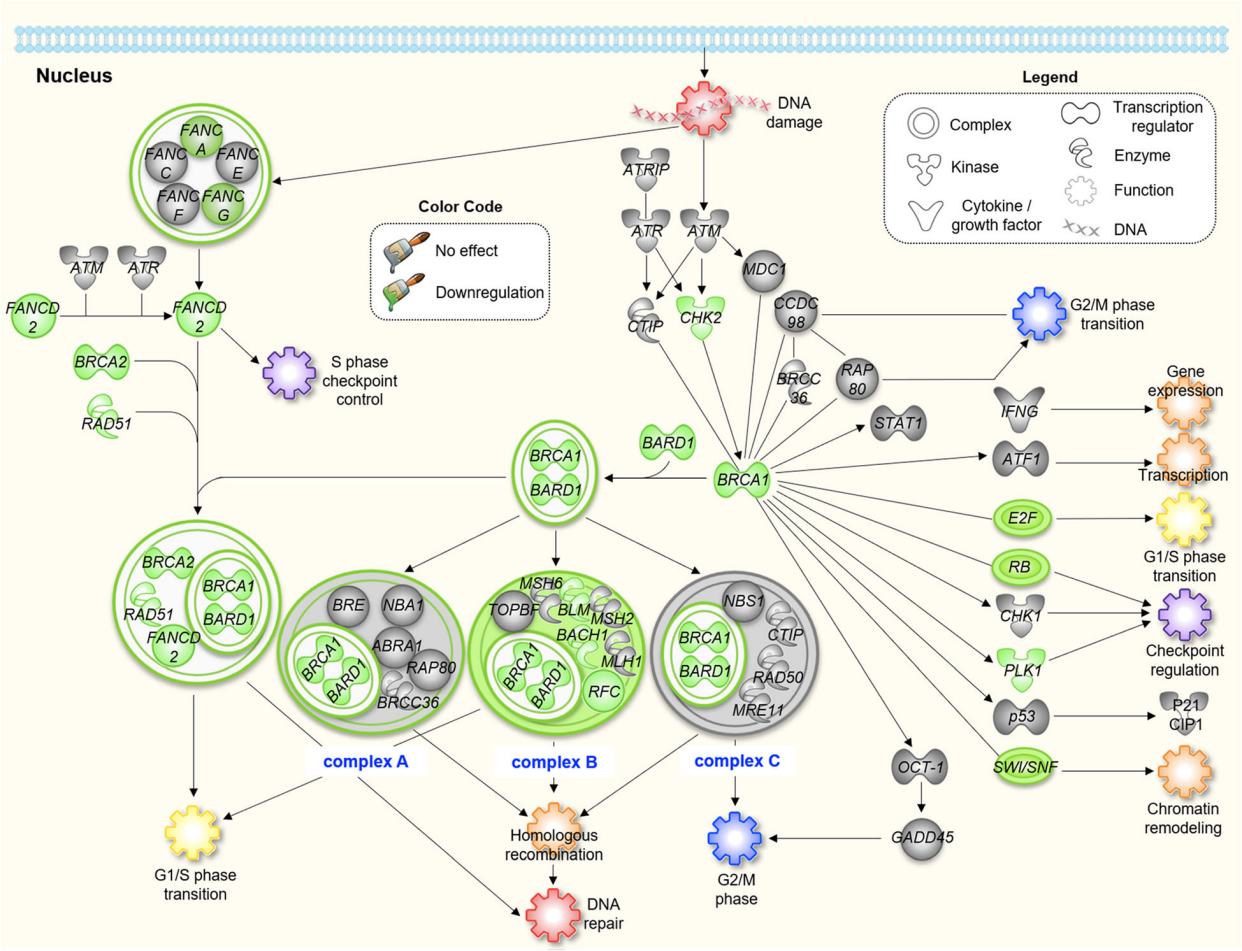
Sal and Das together synergistically inhibited the BRCA1 pathway in the MDA-MB-468 cell line. A schematic diagram showing how the drug combination inhibited the BRAC1 pathway. The drug combination selectively suppressed the genes associated with the formation of complex B within the BRCA1 pathway

Although IPA identified the estrogen-mediated S-phase entry pathway was the most significant pathway modulated (suppressed) by our drug combination, we could not rule out the possibility that other pathways were involved in the synergistic effect. A recent meta-analysis of RNA-seq data from multiple studies revealed that the BRCA1 and DNA damage response pathway was upregulated in breast tumors compared to healthy breast tissues, in addition to the estrogen-mediated S-phase entry pathway [66]. In the present study, IPA identified the BRCA1 and DNA damage response pathway as the fourth most significant pathway modulated by the drug combination (Fig. 4a). The transcription of *BRCA1* is regulated by an upstream early growth response protein 1 (EGR1; encoded by *EGR1*) through binding to the EGR1-binding sequences within the enhancer region of *BRCA1* [67]. Either Sal and Das alone or in combination suppressed EGR1 (data not shown). Functionally, BRCA1 is a central mediator that controls the activities of multiple DNA repair and checkpoint pathways [68]. The protein forms a heterodimer with BRCA associated RING domain protein 1 (BARD1), which is essential for interacting with different transcription regulators to form 3 distinct complexes: BRCA1 A, B, and C complexes (Figs. 7**and S16**) [69]. Complex A is involved in DNA repair via homologous recombination. Complexes B and C regulate cell cycle checkpoint, in addition to repairing damaged DNA. An in-depth analysis of our RNA-seq data uncovered that Sal and Das together synergistically inhibited the BRCA1 and DNA damage response pathway (Figs. 7**and****S16**). Interestingly, the drug combination selectively impaired the pathway predominantly via an inhibition of the BRCA1 B complex. This was evidenced by the downregulation of a majority of the genes associated with the complex formation, including *BACH1* (encodes BRCA1 interacting protein C-terminal helicase), and *BLM* (encodes blood syndrome RecQ like helicase), and *RFC* (replication factor C). Further studies are needed to confirm the drug inhibitory effects at the protein translational level as well as the therapeutic benefits of targeting BRCA1 B complex and causing cell cycle arrest. Finally, a recent study identified nucleolin as the functional binding target of Sal. The drug inhibited the transcription of the nucleolin gene (*NCL*) and led to a suppression of downstream *CD34* gene expression [70], which was also shown in our RNA-seq data.

## Conclusion

In conclusion, we employed RNA-seq to investigate the global transcriptomic changes induced by Sal and Das separately and together in the human TNBC cell line MDA-MB-468. An advantage of using RNA-seq is that it allowed us to revisit the mechanisms of action and identify new therapeutic targets. Consistent with the literatures, Sal and Das suppressed known genes, including *BIRC5*, *CCND1*, *MCL1*, *MYC*, *AXIN2*, *MMP7*, *BMP4*, *BMP5*, and many others that are regulated by the STAT3, Wnt/β-catenin, and hedgehog cell signaling pathways. However, the drug combination did not seem to enhance the suppression of these genes. The drug combination exhibited synergism through horizontal suppression of multiple pathways, leading to a promotion of cell cycle arrest at the G1/S phase partially via the estrogen-mediated S-phase entry pathway, as well as the BRCA1 and DNA damage response pathway. Our studies demonstrated, for the first time, that Sal could downregulate *E2F2*. We also discovered a novel mechanism of the drug to upregulate *ESR2*. Currently, there is a lack of targeted therapy for TNBC. Such a drug-induced ESR2 expression might lead to a novel targeting approach by using a combination of Sal and Tamo for TNBC treatment. However, further studies are required to address the in vivo efficacy and to repurpose/restratify the clinical role endocrine (hormone) therapy for BC treatment.

Many biological processes are regulated by enzyme activities, such as phosphorylation (by kinases) and proteolysis (by proteases), rather than actual gene and protein expression. RNA-seq can provide a comprehensive insight into drug actions, but the information obtained was limited to the transcriptomic level. Further, not all gene expression will translate into proteins leading to quantitative and qualitative divergences between genomic/transcriptomic modulations and their protein counterparts [71]. A recent retrospective and integrated genomic, transcriptomic, and protein analysis identified the transcript and protein products of SPAG5 as biomarkers of chemotherapy sensitivity in estrogen receptor-negative BC [72]. Another study exploited protein expression from reverse-phase protein assay and RNA-seq data to study the functional consequences of PIK3CA mutation leading to the tumorigenesis of HER2^+^/ER^+^ BC [73]. We believe that using a combined multi-omics approach to study drug mechanisms will allow us to identify more specific disease-relevant biomarkers for precision treatment of BC, as well as other cancers in the future.

## Supplementary information

**Additional file 1 Table S1:** Table summarizing the source and the identifier of the reagents and resources used for this study.

**Additional file 2 Table S2:** Table summarizing the number of genes upregulated and downregulated by different drug treatments. MDA-MB-468 cells were treated with the Sal or Das alone, or the drug combination (S + D) for 24 h and 72 h prior to extract the mRNAs for RNA-seq analysis. The experiments were independently performed in quadruplicate.

**Additional file 3 Figure S1:** Human primer sequences used for the RT-qPCR analysis.

**Additional file 4 Figure S2:** Examples of drug combination synergism relying on drug ratio. (A) Chemical structure of doxorubicin (Dox). (B) Cell viability assay of Dox against different human BC cell lines (MDA-MB-468, MDA-MB-231, and MCF-7). The cell viability was first measured 72 h after the drug incubations. (C) The results were then fit into sigmoidal dose response curves for calculating the IC_50_ values. A table summarizing the IC_50_ values of Dox determined using different cell lines. (D) Tables showing the CI values of 2-drug combinations of Dox and Das (Dox + Das) or Sal (Dox + Sal) determined at various drug ratios in MDA-MB-468 cell line. The CI values were determined 72 h after the drug incubations according to the previously described Chou-Talalay method [33]. The CI_95_ represents the specific CI value at 95% of cell growth inhibition. Inset: S = synergistic effect. SS = strongly synergistic effect. (E) Representative simulated plots of the CI values of the drug combinations Dox + Das and Dox + Sal (at different drug ratios) versus the cellular fraction affected (Fa) values. The plots were generated using CompuSyn software. All the experiments were independently performed in triplicate.

**Additional file 5 Figure S3:** Representative computer-simulated plots of the CI values of the drug combination (at different drug ratios) versus the cellular fraction affected (Fa = 1 – the ratio of the drug-treated to the non-treated cell numbers). The plots were generated using the CompuSyn software, based on the cell viability data determined in the human BC cell lines MDA-MB-468, MDA-MB-231 and MCF-7 maintained in (A) cell cultures or (B) tumor spheroids. All the experiments were independently performed in triplicate.

**Additional file 6 Figure S4:** The cytotoxicity of the drug alone or in combination was assessed in the 3 different BC cell lines using Trypan Blue exclusion assay. (A) Representative images of different cancer cells after treatment for 72 h with Sal, Das, or the drug combination at their corresponding IC_50_ concentrations (Fig. 1c). Cells were incubated with Trypan Blue solution (0.4%) for 3 min prior optical imaging. Scale bar is 50 μm. (B) Proportion of dead cell induced by the different drug treatments. Cells stained with Trypan Blue are considered as non-viable. Data were presented as mean ± standard deviation (SD) and statistical differences were analyzed using Student’s *t*-test (*P < 0.05, **P < 0.01).

**Additional file 7 Figure S5:** Representative microscopic images of the BC spheroids incubated for 72 h with the drugs alone or in combination at their corresponding IC_50_ concentrations (Fig. 1h). Scale bar is 200 μm. (B) Evolution of the size of the spheroids following the different drug treatments. Four random diameters were measured on each picture.

**Additional file 8 Figure S6:** Effects of Sal and Das alone or in combination on apoptosis and necroptosis. The proportion of healthy, apoptotic, necrotic, and dead cells was measured by AnV binding and PI uptake using flow cytometry. (A) Plots of PI fluorescence versus AnV fluorescence. MDA-MB-468 cells were incubated with PBS (control), Sal (0.5 μM), Das (15 μM), or the drug combination for 72 h and then stained with AnV-FITC and PI for 10 min prior to FACS analysis. (B) Table showing the quantification of each cell populations following the different drug treatments. All the experiments were performed in triplicate.

**Additional file 9 Figure S7:** Neither Fer-1 nor Nec-1 could rescue the drug induced exposure of phosphatidylserine. (A) Investigation of the effects of ferrostatin-1 (Fer-1) and/or necrostatin-1 (Nec-1) (1 μM of inhibitor content) on the cytotoxicity of our drug combination. MDA-MB-468 cells were treated for 72 h with S + D at different total drug contents (drug ratio fixed at 1:30) in the presence of Fer-1 and/or Nec-1 (1 μM of inhibitor content). The cell viability was evaluated using the CellTiter Glo Luminescent Assay. (B) Representative flow cytometry analysis showed that neither Fer-1 nor Nec-1 changed the proportion of apoptotic (AnV^+^PI^−^)/dead (AnV^+^PI^+^) cells induced by the drug combination. MDA-MB-468 cells were incubated with S + D for 72 h in presence of (Fer-1) and/or (Nec-1) prior to staining with AnV-FITC and PI for FACS analysis. All the experiments were independently performed in triplicate.

**Additional file 10 Figure S8:** Tables summarizing the drug-induced changes in the expressions of targeted genes that are known to be regulated by (A) STAT3, (B) Wnt/β-catenin, and (C) hedgehog pathways. The mRNA expression levels were retrieved from the RNA-seq data, and were presented as a log2 fold change relative to the control cells treated with PBS. The experiments were independently performed in quadruplicate. Note: N.S. = no significant change in the gene expression.

**Additional file 11 Figure S9:** (A) Bar graphs showing the 10 most significant canonical pathways that were modulated in MDA-MB-468 cells 24 h after treatment with Sal (0.5 μM), Das (15 μM), or the drug combination. (B) A table summarizing the changes in expression of the genes associated with the estrogen-mediated S-phase entry pathway. The experiments were performed in quadruplicate. N.S. = no significant change of the gene expression.

**Additional file 12 Figure S10:** Tables summarizing the differential expression of the genes associated with estrogen-mediated S-phase entry pathway in the BC cell line MDA-MB-468, after treatment with PBS, Sal (0.5 μM), Das (15 μM), or the drug combination for 24, 48, and 72 h. Gene expression was determined using a RT-qPCR assay. All the experiments were performed in triplicate. Inset: N.S. = non-significant results; A = antagonistic; Ad = additive; and S = synergistic effect.

**Additional file 13 Figure S11:** Comparison of the RNA-seq and RT-qPCR methods used to quantify the expression of the genes associated with the estrogen-mediated S-phase entry pathway in MDAMB-468 cells. Cells were treated with PBS, Sal (0.5 μM), Das (15 μM), or the drug combination for 72 h prior to the analysis. The differential gene expressions were presented as relative fold changes compared to the expression level of the same gene in cells treated with PBS (control). All the experiments were performed in triplicate.

**Additional file 14 Figure S12:** Tables summarizing the differential expression of the genes associated with estrogen-mediated S-phase entry pathway in human BC cell lines MDA-MB-468, MDA-MB-231, and MCF-7 72 h after treatment with PBS, Sal (0.5 μM), Das (15 μM), or the drug combination. The gene expression was determined using a RT-qPCR assay. All the experiments were performed in triplicate. Inset: N.S. = non-significant results; A = antagonistic; Ad = additive; and S = synergistic effect.

**Additional file 15 Figure S13:** Original western blots used for Fig. 5 and **Fig. S14**. The blots were processed using Image Studio Lite 5.2 software The red boxes indicate the cropped regions used in the representative figures.

**Additional file 16 Figure S14:** (A) Western Blot analysis of ESR2 protein expression in MDA-MB-231 cells after exposure to the PBS, Sal (0.5 μM), Das (15 μM), or in combination for 72 h. GAPDH was used as loading control. The blots were processed and cropped using Image Studio Lite 5.2 software. Full-length blots are available **Fig. S13**. (B) Graph bar showing the expression level of ESR2 protein. The relative protein expression levels were quantified using Image Studio software and normalized to the control cells treated with PBS. All the experiments were performed in triplicate. Data were presented as mean ± standard deviation (SD) and statistical differences were calculated by Student’s *t*-test (*P < 0.05, **P < 0.01). (C) A flow cytometry graph showing an upregulation of ESR2 expression in response to Sal or the drug combination (S + D) treatment. The MDA-MB-231 cells were treated with drugs alone (at the corresponding IC_50_ concentration) or 2-drugs combination prior to incubation with phycoerythrin-labeled anti-ESR2 for FACS analysis. (D) Representative fluorescence microscopic images showing Sal induced ESR2 in MDA-MB-231 cells. Prior to imaging, the cells were incubated with phycoerythrin-labeled anti-ESR2 and DAPI for staining the ESR2 receptors (green) and the nucleus (blue), respectively. Scale bar is 60 μm.

**Additional file 17 Figure S15:** (A) Chemical structure of 4-hydroxytamoxifen (Tamo). (B) Plots of the relative cell viability of MDA-MB-468 cells 72 h after treatment with Das (15 μM), Tamo (1 μM), or the drug combination (D + T).

**Additional file 18 Figure S16:** Sal and Das together synergistically inhibited the BRCA1 pathway in a MDA-MB-468 cell line. A table summarizing the differential gene expression of cells treated with Sal (0.5 μM) or Das (15 μM), or the drug combination. The experiments were performed in quadruplicate. N.S. = no significant change of the gene expression.

## Data Availability

The raw RNA sequencing dataset reported in this paper is available in the Gene Expression Omnibus (GEO) database using the accession number GSE135514 and following the link: https://www.ncbi.nlm.nih.gov/geo/query/acc.cgi?acc=GSE135514. All other resources related to this research work are available upon reasonable request to the lead contact Benedict Law (sbl2004@med.cornell.edu).

## Abbreviations

AKT: Protein kinase B
ALDH1: Aldehyde dehydrogenase 1
BC: Breast cancer
BCL: Binary base call
CCND1: Cyclin D1
CCNE2: Cyclin E2
CDC: Cell division cycle
CDK: Cyclin-dependent kinase
cDNA: Complementary deoxyribonucleic acid
CI: Combination index
CSCs: Cancer stem cells
CYT387: Momelotinib
DAPI: 4′,6-Diamidino-2-phenylindole
Das: Dasatinib
DCF-DA: 2′,7′-Dichlorofluorescein diacetate
DNA: Deoxyribonucleic acid
Dox: Doxorubicin
E2F: E2F transcription factor.
EGFR: Epidermal growth factor receptor
EGR: Early growth response
ER: Estrogen receptor
ESR2: Estrogen receptor β
FACS: Fluorescence-activated cell sorting
FBS: Fetal bovine serum
FITC: Fluorescein isothiocyanate
FPKM: Fragments per kilobase of exon model per million
GAPDH: Glyceraldehyde 3-phosphate dehydrogenase
GEO: Gene expression omnibus
GFP: Green fluorescent protein
HER2: Human epidermal growth factor receptor 2
IC_50_: Half maximal inhibitory concentration
IPA: Ingenuity pathway analysis software
iROS: Intracellular reactive oxygen species
JAK: Janus kinase
JNK: Jun amino-terminal kinase
MAPK: Mitogen-activated protein kinase
M-MLV: Moloney murine leukemia virus
mRNA: Messenger ribonucleic acid
mTOR: Mammalian target of rapamycin
NCBI: National center for biotechnology information
NCL: Nucleolin gene
N.S.: non-significant
P_adj_: Adjusted *p* value
PBS: Phosphate buffered saline
PI: Propidium iodide
PI3K: Phosphoinositide 3-kinase
RBL1: Retinoblastoma-like 1
RNA-seq: Ribonucleic acid sequencing
RT-qPCR: Reverse transcription-quantitative polymerase chain reaction
S + D: Salinomycin + dasatinib
Sal: Salinomycin
SD: Standard deviation
shRNA: Small hairpin ribonucleic acid
SKP: S-phase kinase associated protein
STAT: Signal transducer and activator of transcription
Tamo: 4-Hydroxytamoxifen
TBST: Tris buffer saline containing Tween
TNBC: Triple negative breast cancer
