# Supplementary material for: Transcriptomic insight into salinomycin mechanisms in breast cancer cell lines: synergistic effects with dasatinib and induction of estrogen receptor β
Source: BMC Cancer. 2020 Jul 16;20:661. doi: 10.1186/s12885-020-07134-3 (PMC7364656; doi:10.1186/s12885-020-07134-3)
Supplement: Supplementary file 1 — Additional file 1 Table S1: Table summarizing the source and the identifier of the reagents and resources used for this study. [file 12885_2020_7134_MOESM1_ESM.docx]

| REAGENT or RESOURCE | SOURCE | IDENTIFIER |
| --- | --- | --- |
| **Chemicals** | | |
| Doxorubicin | LC Laboratories | Cat# D-4000 |
| Salinomycin | Cayman Chemical | Cat# 13579 |
| Dasatinib | LC Laboratories | Cat# D-3307 |
| Ferrostatin-1 (Fer-1) | Sigma Aldrich | Cat# SML0583-5MG |
| Necrostatin-1 (Nec-1) | Sigma Aldrich | Cat# N9037-10MG |
| Trypan Blue Stain (0.4%) | ThermoFisher Scientific | Cat# T10282 |
| 2’,7’-dichlorofluorescein diacetate (DCF -DA) | Sigma Aldrich | Cat# D6883-50MG |
| 4’,6-diamidino-2-phenylindole (DAPI) | Life Technologies | Cat# D1306 |
| PowerUp SYBR Green Master Mix | Applied Biosystems | Cat# A25742 |
| Lipofectamine RNAiMAX | ThermoFisher Scientific | Cat# 13778075 |
| RIPA Lysis Buffer | Santa Cruz Biotechnology | Cat# sc- 24948 |
| Matrigel basement membrane matrix | Corning | Cat# 354248 |
| 4-hydroxytamoxifen | Sigma Aldrich | Cat# 579002-5MG |
| **Kits** | | |
| Propidium iodide flow cytometry kit | Abcam | Cat# ab139418 |
| CellTiter-Glo 2.0 Cell viability assay | Promega | Cat# G9242 |
| CellTiter-Glo 3D Cell viability assay | Promega | Cat# G9683 |
| Annexin V-FITC apoptosis staining/detection kit | Abcam | Cat# ab14085 |
| RNeasy Mini Kit | Qiagen | Cat# 74104 |
| M-MLV reverse transcriptase reagent kit | Promega | Cat# M1705 |
| microBCA assay | ThermoFisher Scientific | Cat# 23235 |
| **Experimental models: Human Cell lines** | | |
| MDA-MB-468 | ATCC | Cat# HTB-132 |
| MDA-MB-231 | ATCC | Cat# HTB-26 |
| MCF-7 | ATCC | Cat# HTB-22 |
| **siRNA oligonucleotides** |  |  |
| Silencer Select Negative Control 1 siRNA | ThermoFisher Scientific | Cat# 4390843 |
| Silencer Select Negative Control 2 siRNA | ThermoFisher Scientific | Cat# 4390846 |
| Silencer Select ESR2 siRNA | ThermoFisher Scientific | Cat# 4392420 assay ID# s4826 |
| Silencer Select ESR2 siRNA | ThermoFisher Scientific | Cat# 4392420 assay ID# s4827 |
| Silencer Select ESR2 siRNA | ThermoFisher Scientific | Cat# 4392420 assay ID# s4828 |
| **Primary and Secondary Monoclonal Antibodies** | | |
| Anti-cyclin D1 (SP4) | ThermoFisher Scientific | Cat# MA5-16356 |
| Anti-cyclin E2 (E142) | Abcam | Cat# ab32103 |
| Anti-E2F2 (EPR8622) | Abcam | Cat# ab138515 |
| Anti-estrogen receptor beta | Abcam | Cat# ab3576 |
| Anti-GAPDH (EPR16891) | Abcam | Cat# ab181602 |
| Anti-β-actin (SP124) | Abcam | Cat# ab115777 |
| Horseradish peroxidase-conjugate goat anti-rabbit IgG (H+L) | ThermoFisher Scientific | Cat# 31460 |
| Anti-estrogen receptor beta antibody (Phycoerythrin) | Abcam | Cat# ab205541 |
| **Human primers** | | |
| All primers used in this study | Integrated DNA Technologies | N/A |
| **Software** | | |
| Compusyn | ComboSyn, Inc. | <http://www.combosyn.com/> |
| GraphPad Prism 6.0 | GraphPad | <https://www.graphpad.com/scientific-software/prism/> |
| Ingenuity Pathway Analysis IPA 4.0 | Qiagen | <http://www.quiagenbioinformatics.com/produ>  cts/ingenuity-pathway-analysis/ |
| Kaluza | Bruker | N/A |
| National Center for Biotechnology Information (NCBI) | NIH | http://www.ncbi.nlm.nih.gov/ |
| Image Studio | Li-cor | http://www.licor.com/bio/image-studio-lite/ |
| **Others** | | |
| 8-well chamber slides | Ibidi | Cat# 80821 |
| Spheroid microplate, 96-well black with clear round bottom, ultra low attachment | Corning | Cat# 4520 |
| NuPAGE™ 4-12% Bis-Tris Gel | Invitrogen | Cat# NP0321BOX |
| iBlot gel transfer stacks PVDF, mini | Invitrogen | Cat# IB401002 |
