# Supplementary material for: Transcriptomic insight into salinomycin mechanisms in breast cancer cell lines: synergistic effects with dasatinib and induction of estrogen receptor β
Source: BMC Cancer. 2020 Jul 16;20:661. doi: 10.1186/s12885-020-07134-3 (PMC7364656; doi:10.1186/s12885-020-07134-3)
Supplement: Supplementary file 2 — Additional file 2 Table S2: Table summarizing the number of genes upregulated and downregulated by different drug treatments. MDA-MB-468 cells were treated with the Sal or Das alone, or the drug combination (S + D) for 24 h and 72 h prior to extract the mRNAs for RNA-seq analysis. The experiments were independently performed in quadruplicate. [file 12885_2020_7134_MOESM2_ESM.docx]

|  | **24 h** | | | **72 h** | | |
| --- | --- | --- | --- | --- | --- | --- |
| Number  of genes | **Sal** | **Das** | **S + D** | **Sal** | **Das** | **S + D** |
| ***Down*** | 0 | 154 | 442 | 1487 | 355 | 1669 |
| ***Up*** | 95 | 188 | 417 | 1466 | 341 | 1559 |
