# Supplementary figures and images for: Transcriptomic insight into salinomycin mechanisms in breast cancer cell lines: synergistic effects with dasatinib and induction of estrogen receptor β
Source: BMC Cancer. 2020 Jul 16;20:661. doi: 10.1186/s12885-020-07134-3 (PMC7364656; doi:10.1186/s12885-020-07134-3)

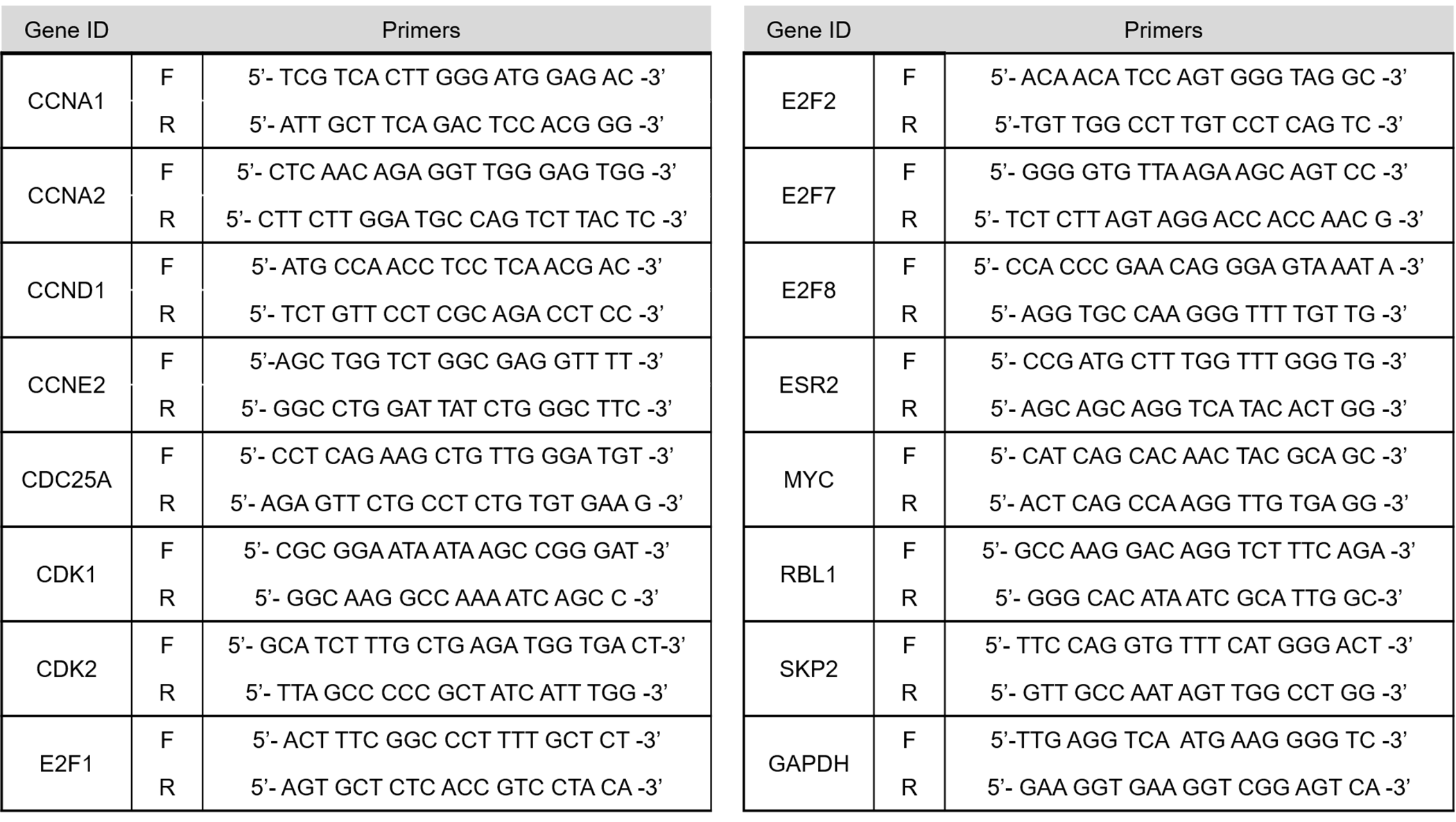

Supplement: Supplementary file 3 — Additional file 3 Figure S1: Human primer sequences used for the RT-qPCR analysis. [file 12885_2020_7134_MOESM3_ESM.tif]

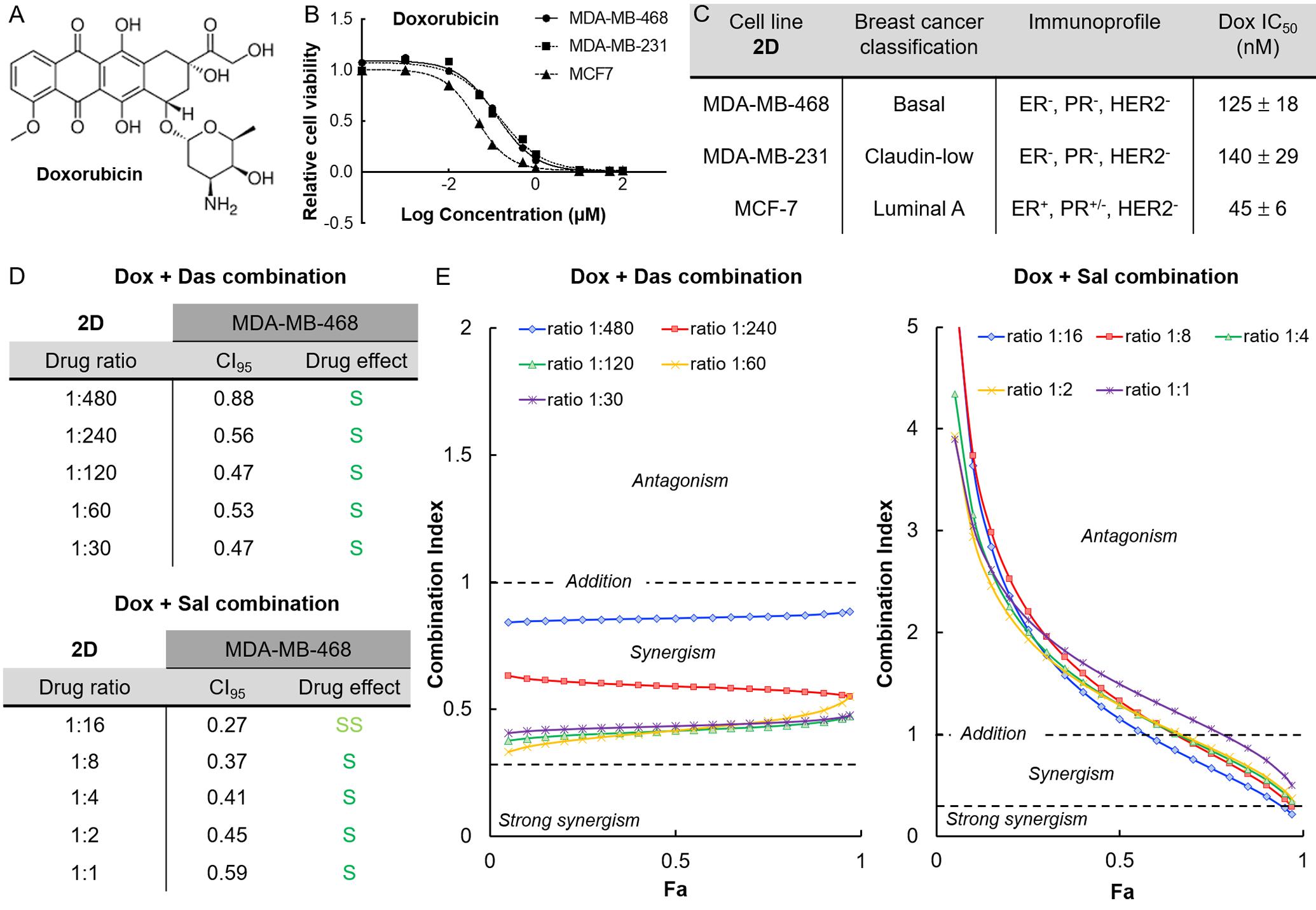

Supplement: Supplementary file 4 — Additional file 4 Figure S2: Examples of drug combination synergism relying on drug ratio. (A) Chemical structure of doxorubicin (Dox). (B) Cell viability assay of Dox against different human BC cell lines (MDA-MB-468, MDA-MB-231, and MCF-7). The cell viability was first measured 72 h after the drug incubations. (C) The results were then fit into sigmoidal dose response curves for calculating the IC50 values. A table summarizing the IC50 values of Dox determined using different cell lines. (D) Tables showing the CI values of 2-drug combinations of Dox and Das (Dox + Das) or Sal (Dox + Sal) determined at various drug ratios in MDA-MB-468 cell line. The CI values were determined 72 h after the drug incubations according to the previously described Chou-Talalay method [33]. The CI95 represents the specific CI value at 95% of cell growth inhibition. Inset: S = synergistic effect. SS = strongly synergistic effect. (E) Representative simulated plots of the CI values of the drug combinations Dox + Das and Dox + Sal (at different drug ratios) versus the cellular fraction affected (Fa) values. The plots were generated using CompuSyn software. All the experiments were independently performed in triplicate. [file 12885_2020_7134_MOESM4_ESM.tif]

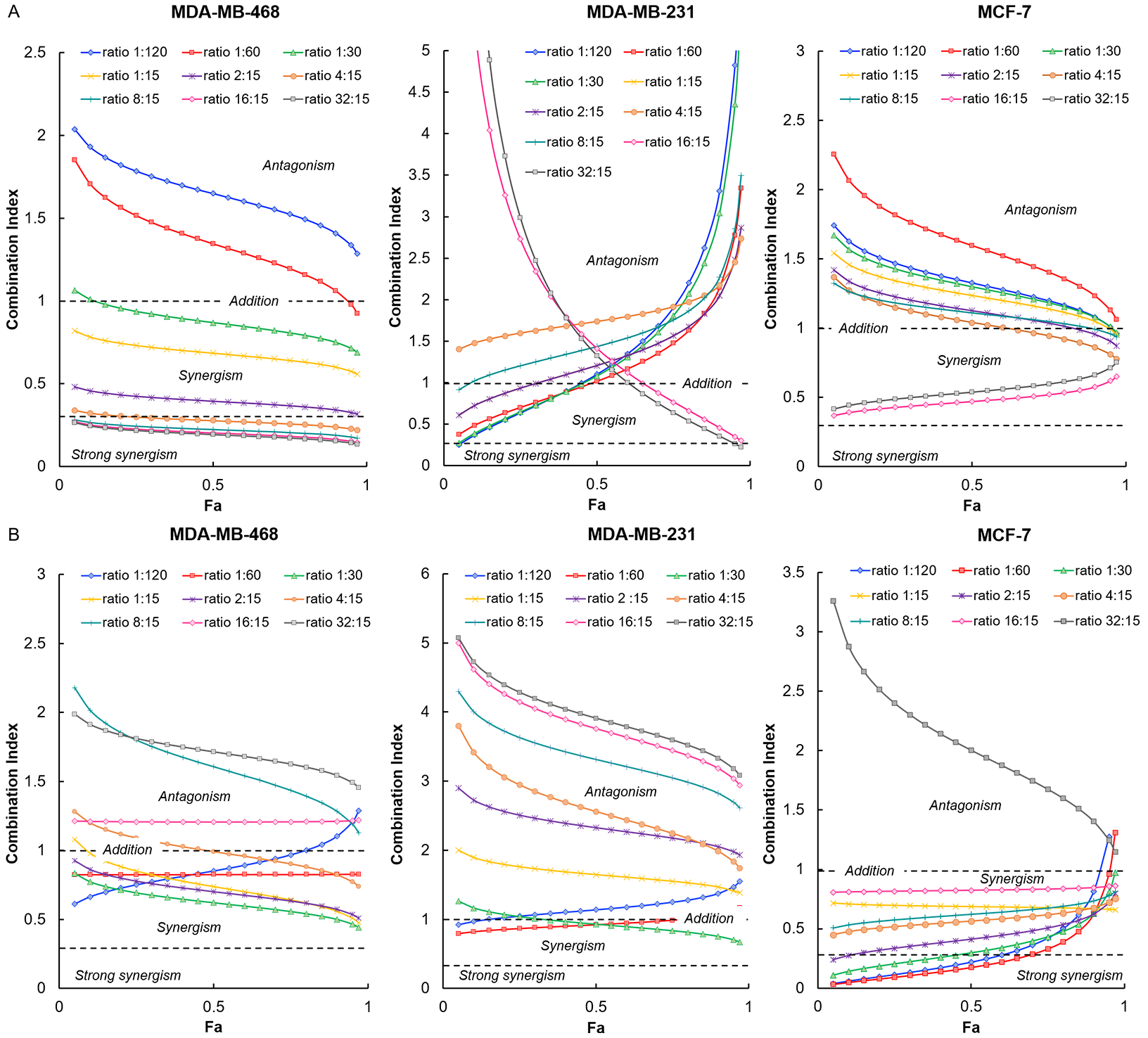

Supplement: Supplementary file 5 — Additional file 5 Figure S3: Representative computer-simulated plots of the CI values of the drug combination (at different drug ratios) versus the cellular fraction affected (Fa = 1 – the ratio of the drug-treated to the non-treated cell numbers). The plots were generated using the CompuSyn software, based on the cell viability data determined in the human BC cell lines MDA-MB-468, MDA-MB-231 and MCF-7 maintained in (A) cell cultures or (B) tumor spheroids. All the experiments were independently performed in triplicate. [file 12885_2020_7134_MOESM5_ESM.tif]

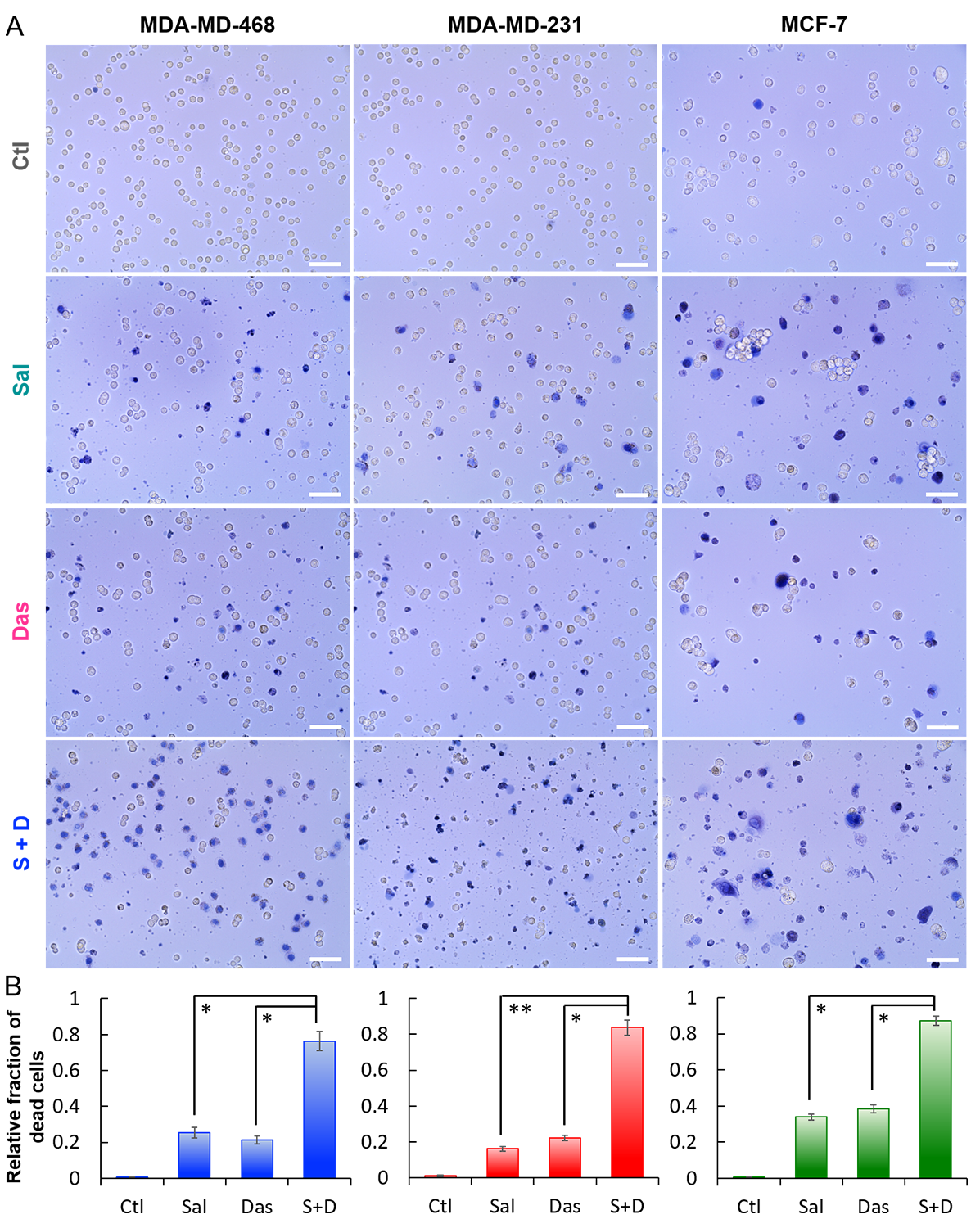

Supplement: Supplementary file 6 — Additional file 6 Figure S4: The cytotoxicity of the drug alone or in combination was assessed in the 3 different BC cell lines using Trypan Blue exclusion assay. (A) Representative images of different cancer cells after treatment for 72 h with Sal, Das, or the drug combination at their corresponding IC50 concentrations (Fig. 1c). Cells were incubated with Trypan Blue solution (0.4%) for 3 min prior optical imaging. Scale bar is 50 μm. (B) Proportion of dead cell induced by the different drug treatments. Cells stained with Trypan Blue are considered as non-viable. Data were presented as mean ± standard deviation (SD) and statistical differences were analyzed using Student’s t-test (*P < 0.05, **P < 0.01). [file 12885_2020_7134_MOESM6_ESM.tif]

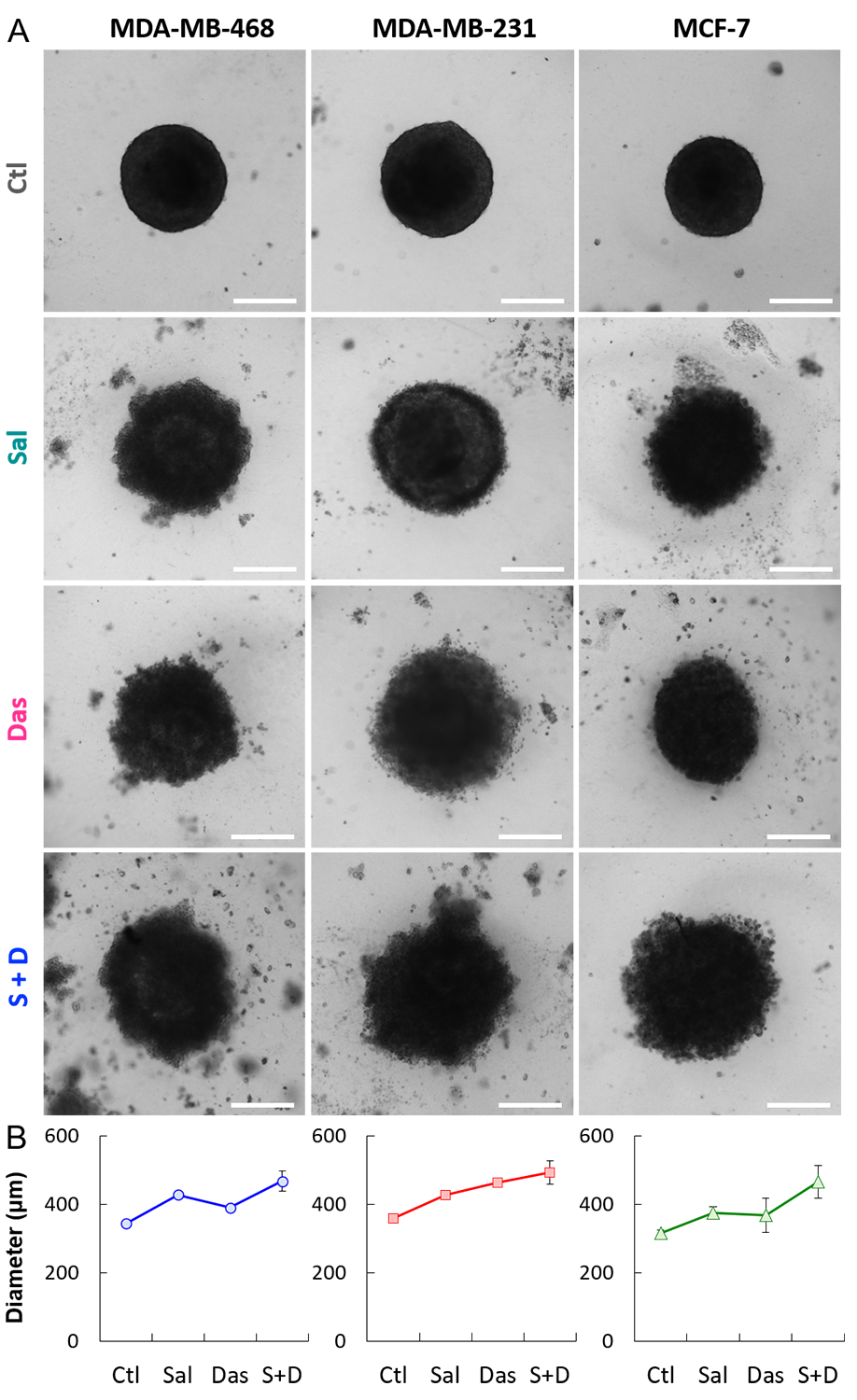

Supplement: Supplementary file 7 — Additional file 7 Figure S5: Representative microscopic images of the BC spheroids incubated for 72 h with the drugs alone or in combination at their corresponding IC50 concentrations (Fig. 1h). Scale bar is 200 μm. (B) Evolution of the size of the spheroids following the different drug treatments. Four random diameters were measured on each picture. [file 12885_2020_7134_MOESM7_ESM.tif]

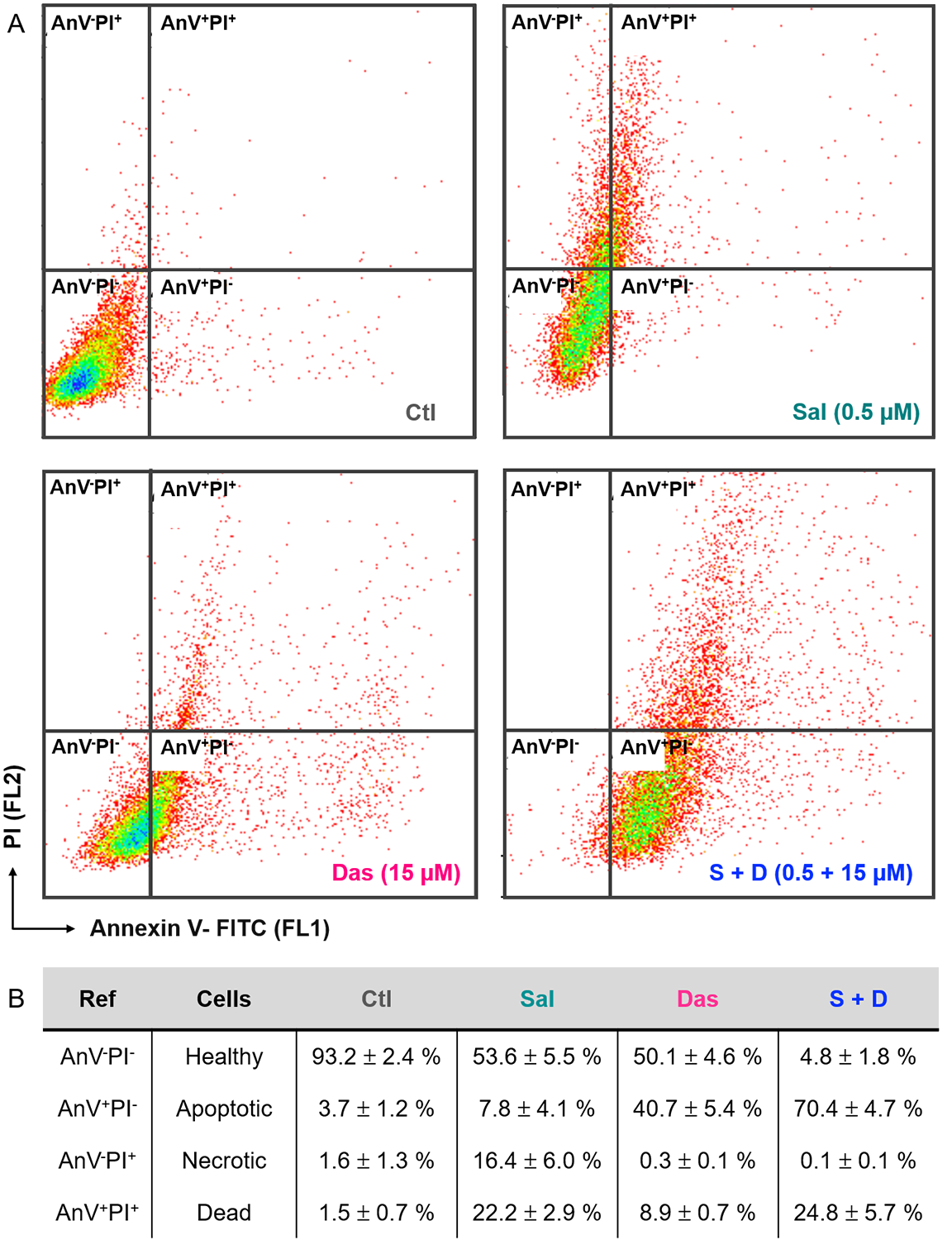

Supplement: Supplementary file 8 — Additional file 8 Figure S6: Effects of Sal and Das alone or in combination on apoptosis and necroptosis. The proportion of healthy, apoptotic, necrotic, and dead cells was measured by AnV binding and PI uptake using flow cytometry. (A) Plots of PI fluorescence versus AnV fluorescence. MDA-MB-468 cells were incubated with PBS (control), Sal (0.5 μM), Das (15 μM), or the drug combination for 72 h and then stained with AnV-FITC and PI for 10 min prior to FACS analysis. (B) Table showing the quantification of each cell populations following the different drug treatments. All the experiments were performed in triplicate. [file 12885_2020_7134_MOESM8_ESM.tif]

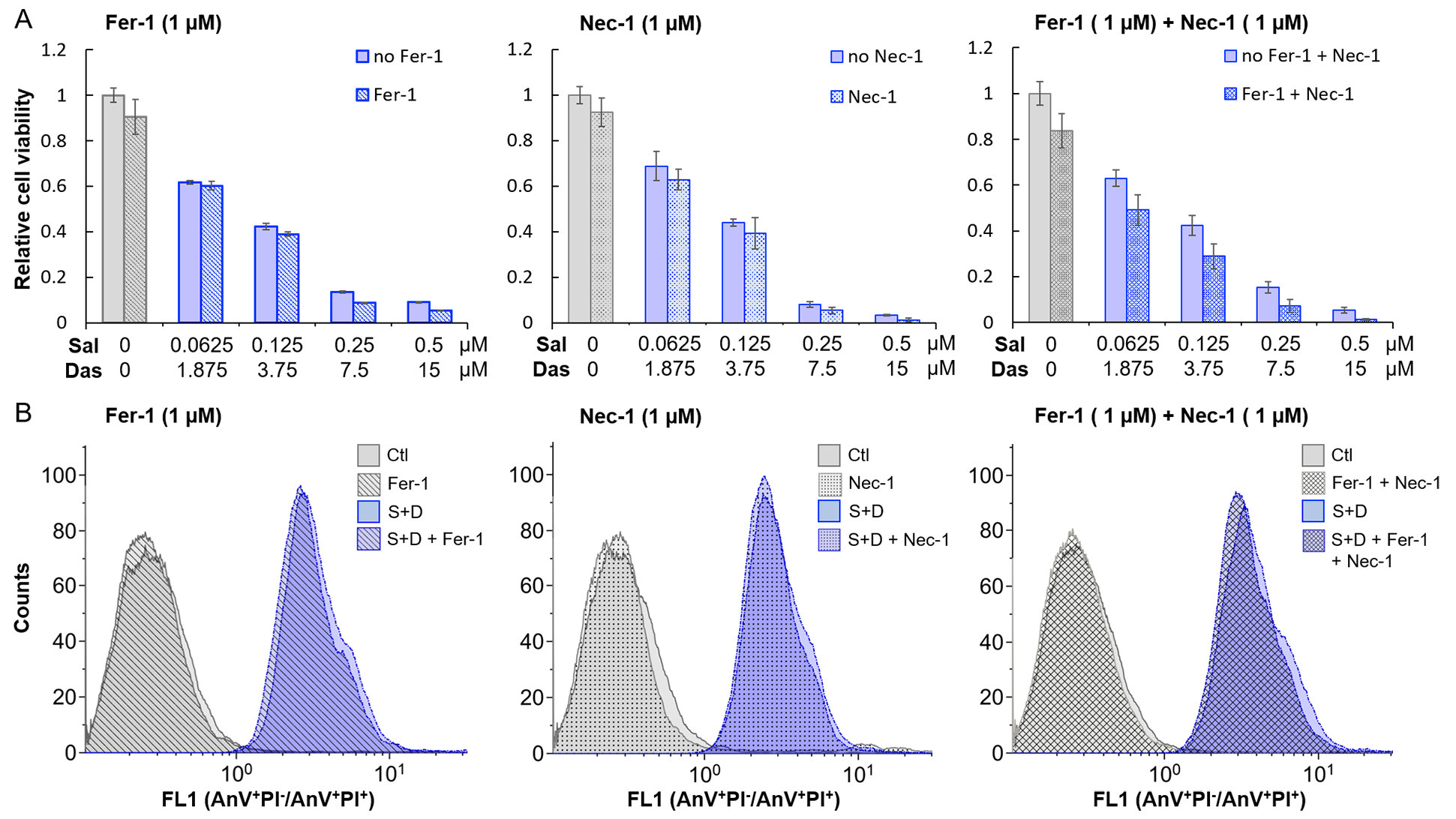

Supplement: Supplementary file 9 — Additional file 9 Figure S7: Neither Fer-1 nor Nec-1 could rescue the drug induced exposure of phosphatidylserine. (A) Investigation of the effects of ferrostatin-1 (Fer-1) and/or necrostatin-1 (Nec-1) (1 μM of inhibitor content) on the cytotoxicity of our drug combination. MDA-MB-468 cells were treated for 72 h with S + D at different total drug contents (drug ratio fixed at 1:30) in the presence of Fer-1 and/or Nec-1 (1 μM of inhibitor content). The cell viability was evaluated using the CellTiter Glo Luminescent Assay. (B) Representative flow cytometry analysis showed that neither Fer-1 nor Nec-1 changed the proportion of apoptotic (AnV+PI−)/dead (AnV+PI+) cells induced by the drug combination. MDA-MB-468 cells were incubated with S + D for 72 h in presence of (Fer-1) and/or (Nec-1) prior to staining with AnV-FITC and PI for FACS analysis. All the experiments were independently performed in triplicate. [file 12885_2020_7134_MOESM9_ESM.tif]

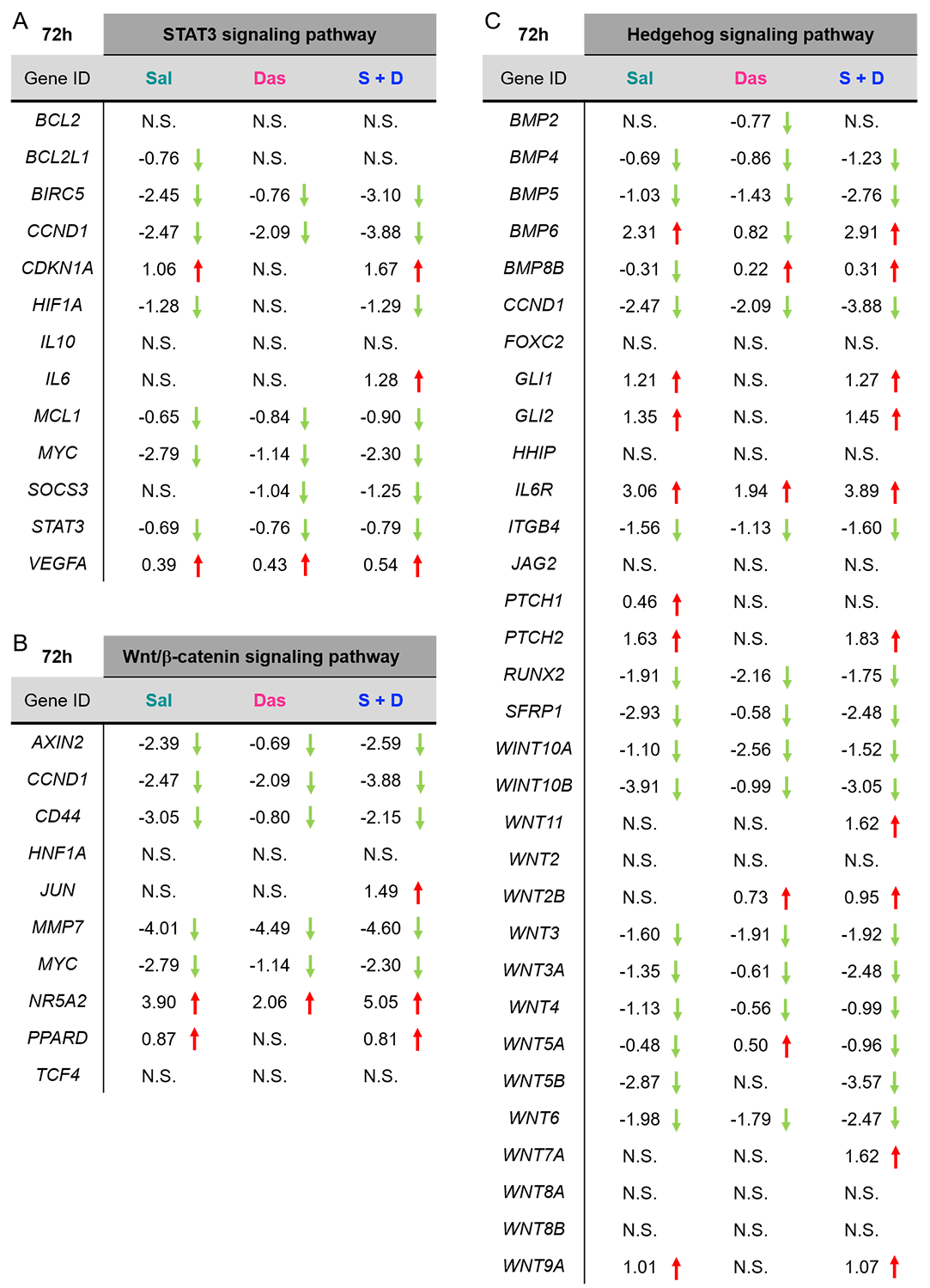

Supplement: Supplementary file 10 — Additional file 10 Figure S8: Tables summarizing the drug-induced changes in the expressions of targeted genes that are known to be regulated by (A) STAT3, (B) Wnt/β-catenin, and (C) hedgehog pathways. The mRNA expression levels were retrieved from the RNA-seq data, and were presented as a log2 fold change relative to the control cells treated with PBS. The experiments were independently performed in quadruplicate. Note: N.S. = no significant change in the gene expression. [file 12885_2020_7134_MOESM10_ESM.tif]

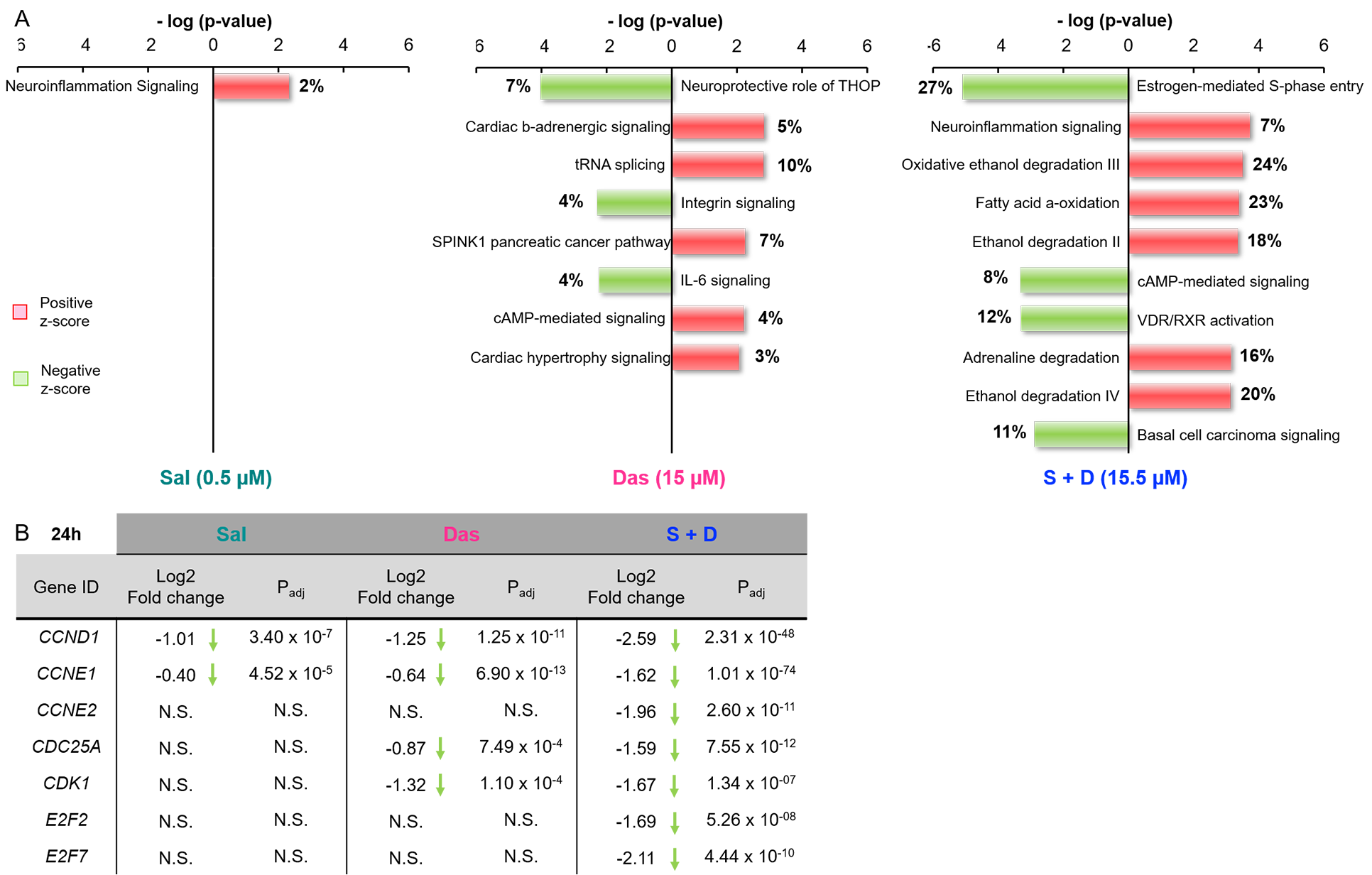

Supplement: Supplementary file 11 — Additional file 11 Figure S9: (A) Bar graphs showing the 10 most significant canonical pathways that were modulated in MDA-MB-468 cells 24 h after treatment with Sal (0.5 μM), Das (15 μM), or the drug combination. (B) A table summarizing the changes in expression of the genes associated with the estrogen-mediated S-phase entry pathway. The experiments were performed in quadruplicate. N.S. = no significant change of the gene expression. [file 12885_2020_7134_MOESM11_ESM.tif]

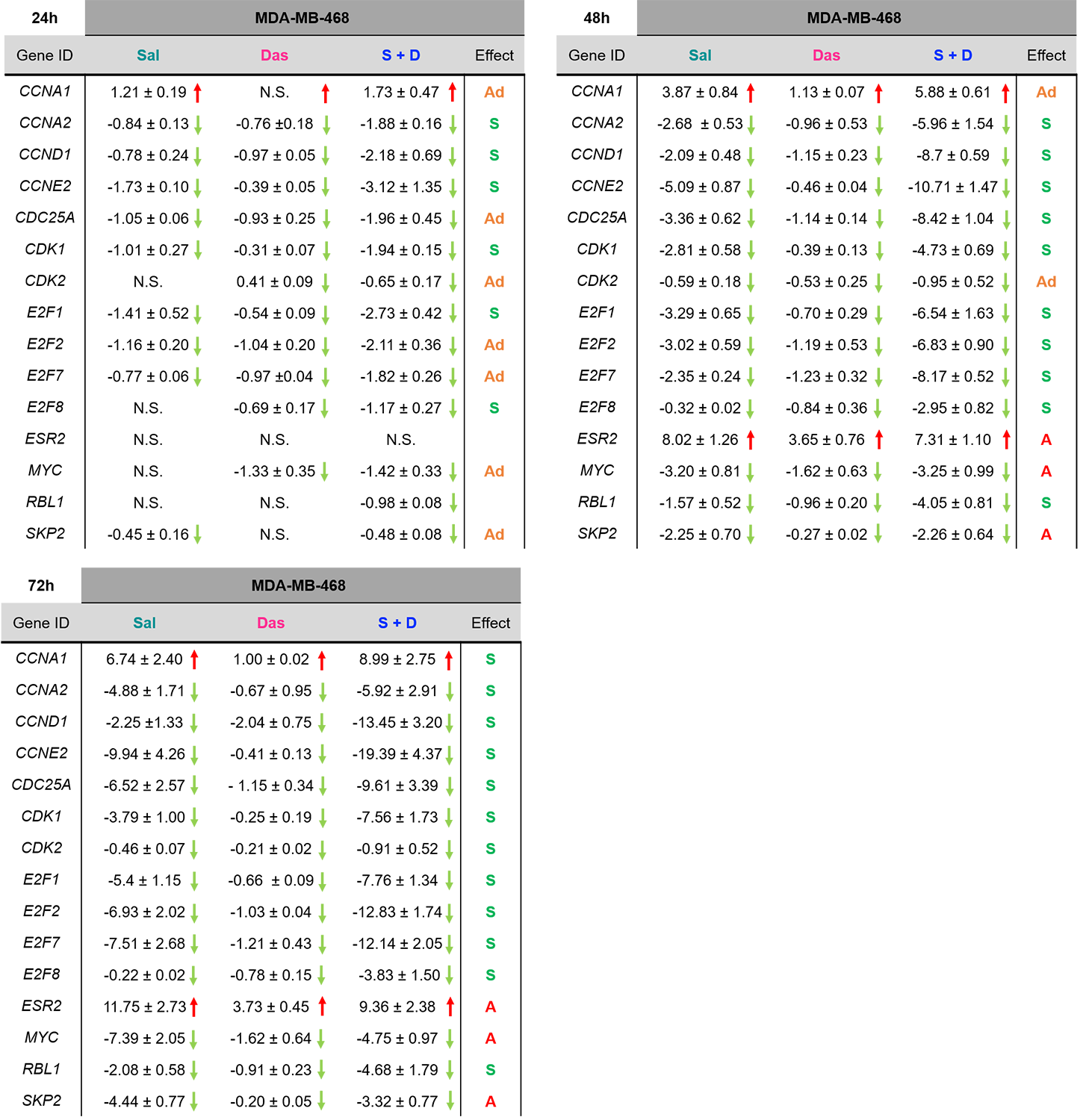

Supplement: Supplementary file 12 — Additional file 12 Figure S10: Tables summarizing the differential expression of the genes associated with estrogen-mediated S-phase entry pathway in the BC cell line MDA-MB-468, after treatment with PBS, Sal (0.5 μM), Das (15 μM), or the drug combination for 24, 48, and 72 h. Gene expression was determined using a RT-qPCR assay. All the experiments were performed in triplicate. Inset: N.S. = non-significant results; A = antagonistic; Ad = additive; and S = synergistic effect. [file 12885_2020_7134_MOESM12_ESM.tif]

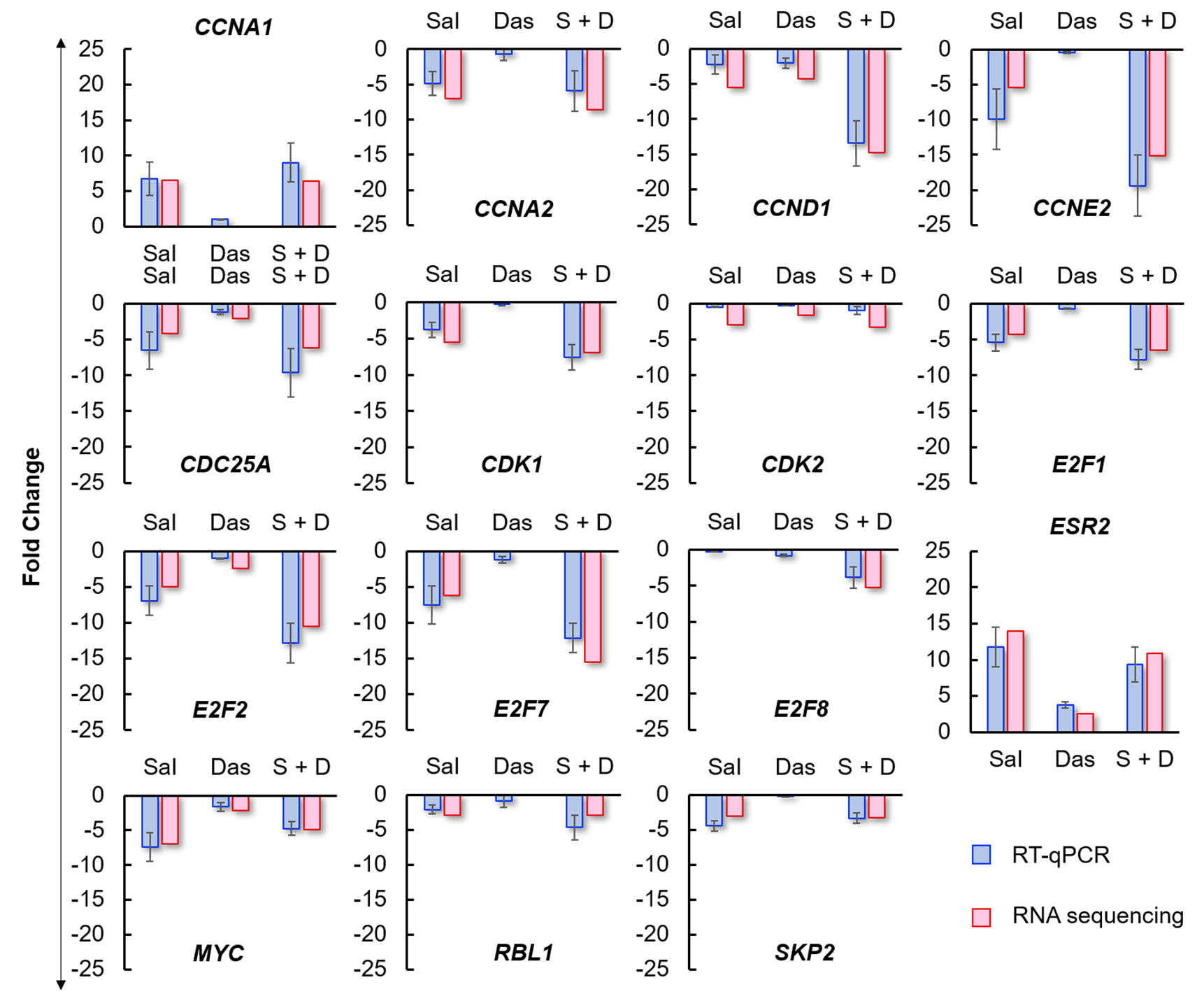

Supplement: Supplementary file 13 — Additional file 13 Figure S11: Comparison of the RNA-seq and RT-qPCR methods used to quantify the expression of the genes associated with the estrogen-mediated S-phase entry pathway in MDAMB-468 cells. Cells were treated with PBS, Sal (0.5 μM), Das (15 μM), or the drug combination for 72 h prior to the analysis. The differential gene expressions were presented as relative fold changes compared to the expression level of the same gene in cells treated with PBS (control). All the experiments were performed in triplicate. [file 12885_2020_7134_MOESM13_ESM.tif]

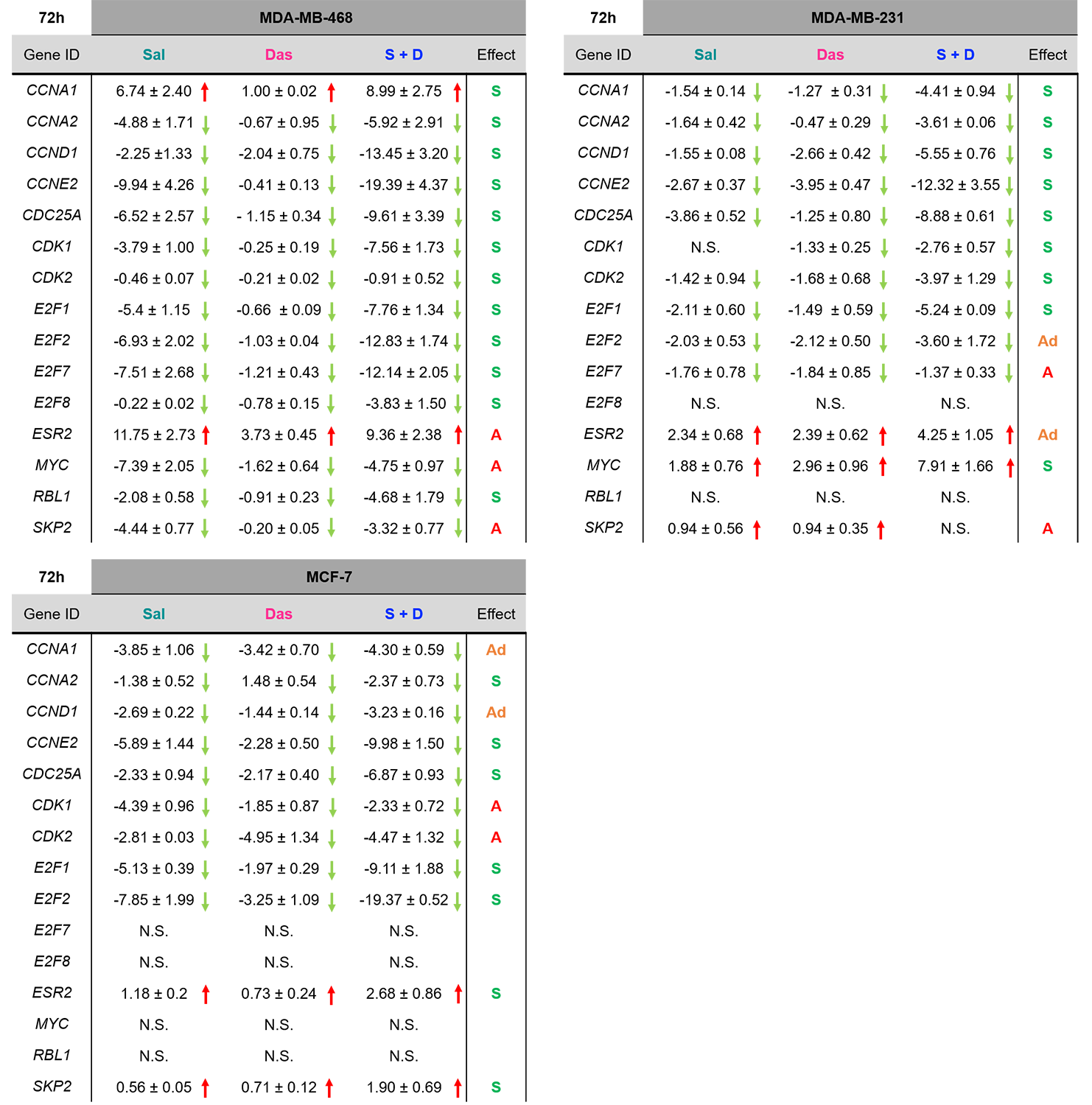

Supplement: Supplementary file 14 — Additional file 14 Figure S12: Tables summarizing the differential expression of the genes associated with estrogen-mediated S-phase entry pathway in human BC cell lines MDA-MB-468, MDA-MB-231, and MCF-7 72 h after treatment with PBS, Sal (0.5 μM), Das (15 μM), or the drug combination. The gene expression was determined using a RT-qPCR assay. All the experiments were performed in triplicate. Inset: N.S. = non-significant results; A = antagonistic; Ad = additive; and S = synergistic effect. [file 12885_2020_7134_MOESM14_ESM.tif]

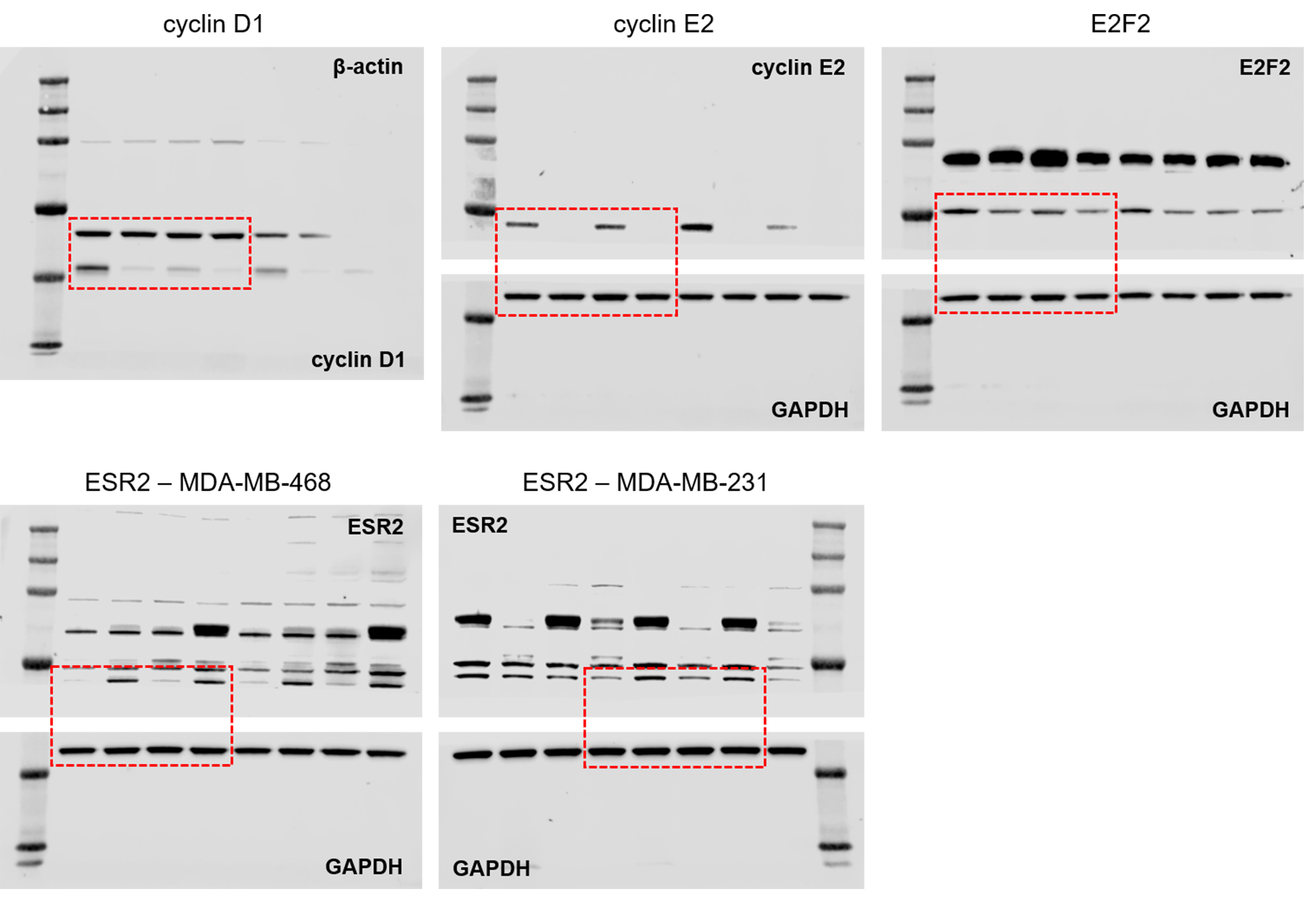

Supplement: Supplementary file 15 — Additional file 15 Figure S13: Original western blots used for Fig. 5 and Fig. S14. The blots were processed using Image Studio Lite 5.2 software The red boxes indicate the cropped regions used in the representative figures. [file 12885_2020_7134_MOESM15_ESM.tif]

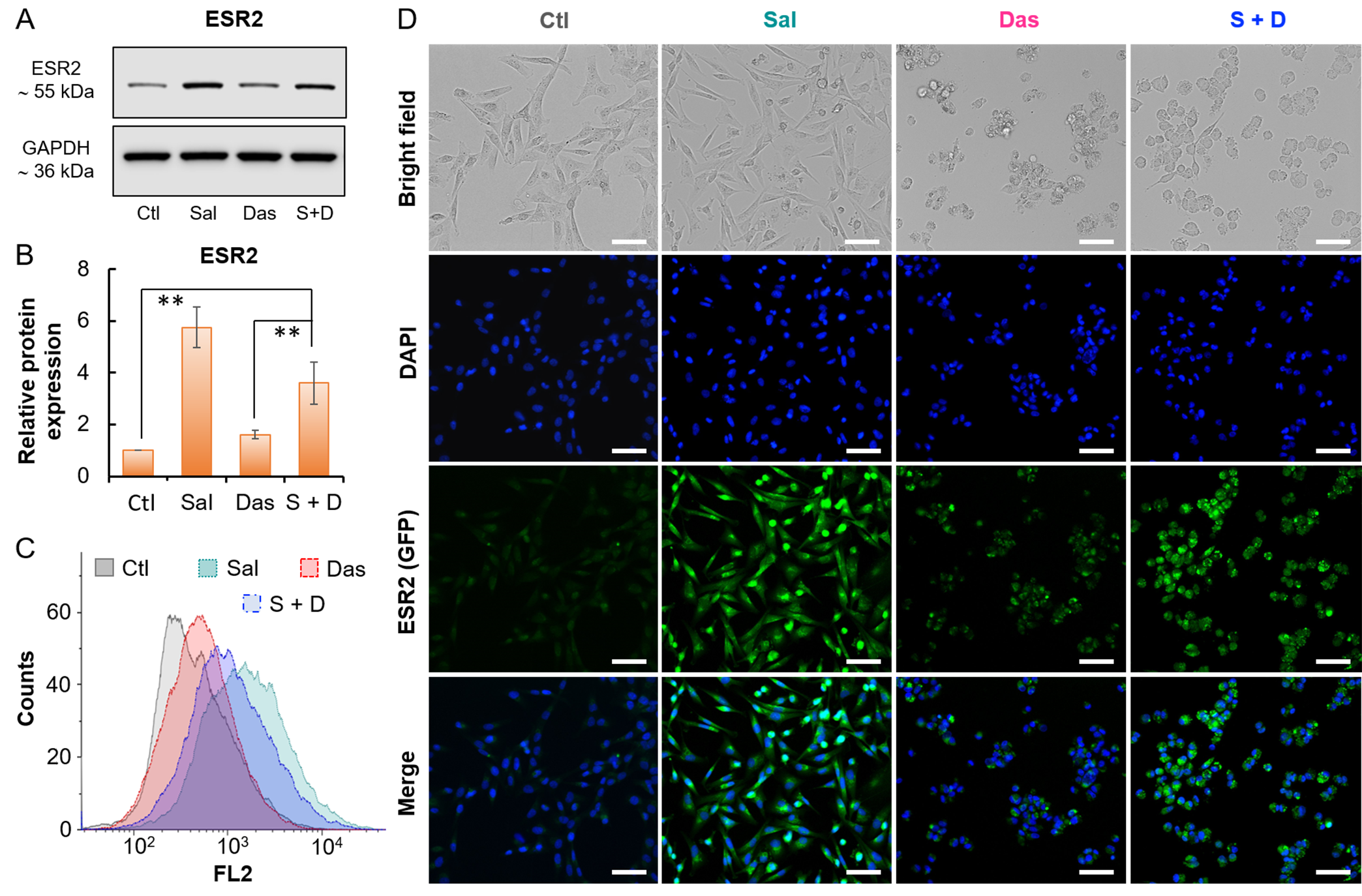

Supplement: Supplementary file 16 — Additional file 16 Figure S14: (A) Western Blot analysis of ESR2 protein expression in MDA-MB-231 cells after exposure to the PBS, Sal (0.5 μM), Das (15 μM), or in combination for 72 h. GAPDH was used as loading control. The blots were processed and cropped using Image Studio Lite 5.2 software. Full-length blots are available Fig. S13. (B) Graph bar showing the expression level of ESR2 protein. The relative protein expression levels were quantified using Image Studio software and normalized to the control cells treated with PBS. All the experiments were performed in triplicate. Data were presented as mean ± standard deviation (SD) and statistical differences were calculated by Student’s t-test (*P < 0.05, **P < 0.01). (C) A flow cytometry graph showing an upregulation of ESR2 expression in response to Sal or the drug combination (S + D) treatment. The MDA-MB-231 cells were treated with drugs alone (at the corresponding IC50 concentration) or 2-drugs combination prior to incubation with phycoerythrin-labeled anti-ESR2 for FACS analysis. (D) Representative fluorescence microscopic images showing Sal induced ESR2 in MDA-MB-231 cells. Prior to imaging, the cells were incubated with phycoerythrin-labeled anti-ESR2 and DAPI for staining the ESR2 receptors (green) and the nucleus (blue), respectively. Scale bar is 60 μm. [file 12885_2020_7134_MOESM16_ESM.tif]

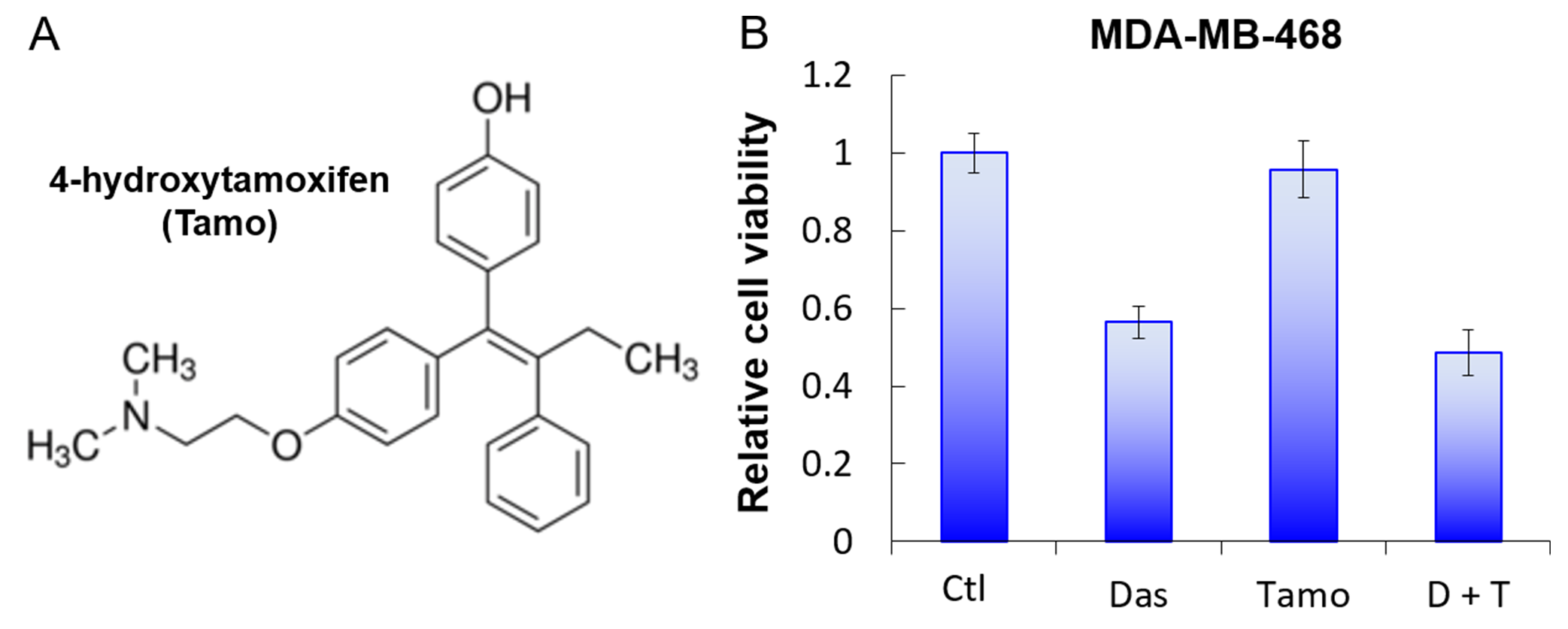

Supplement: Supplementary file 17 — Additional file 17 Figure S15: (A) Chemical structure of 4-hydroxytamoxifen (Tamo). (B) Plots of the relative cell viability of MDA-MB-468 cells 72 h after treatment with Das (15 μM), Tamo (1 μM), or the drug combination (D + T). [file 12885_2020_7134_MOESM17_ESM.tif]

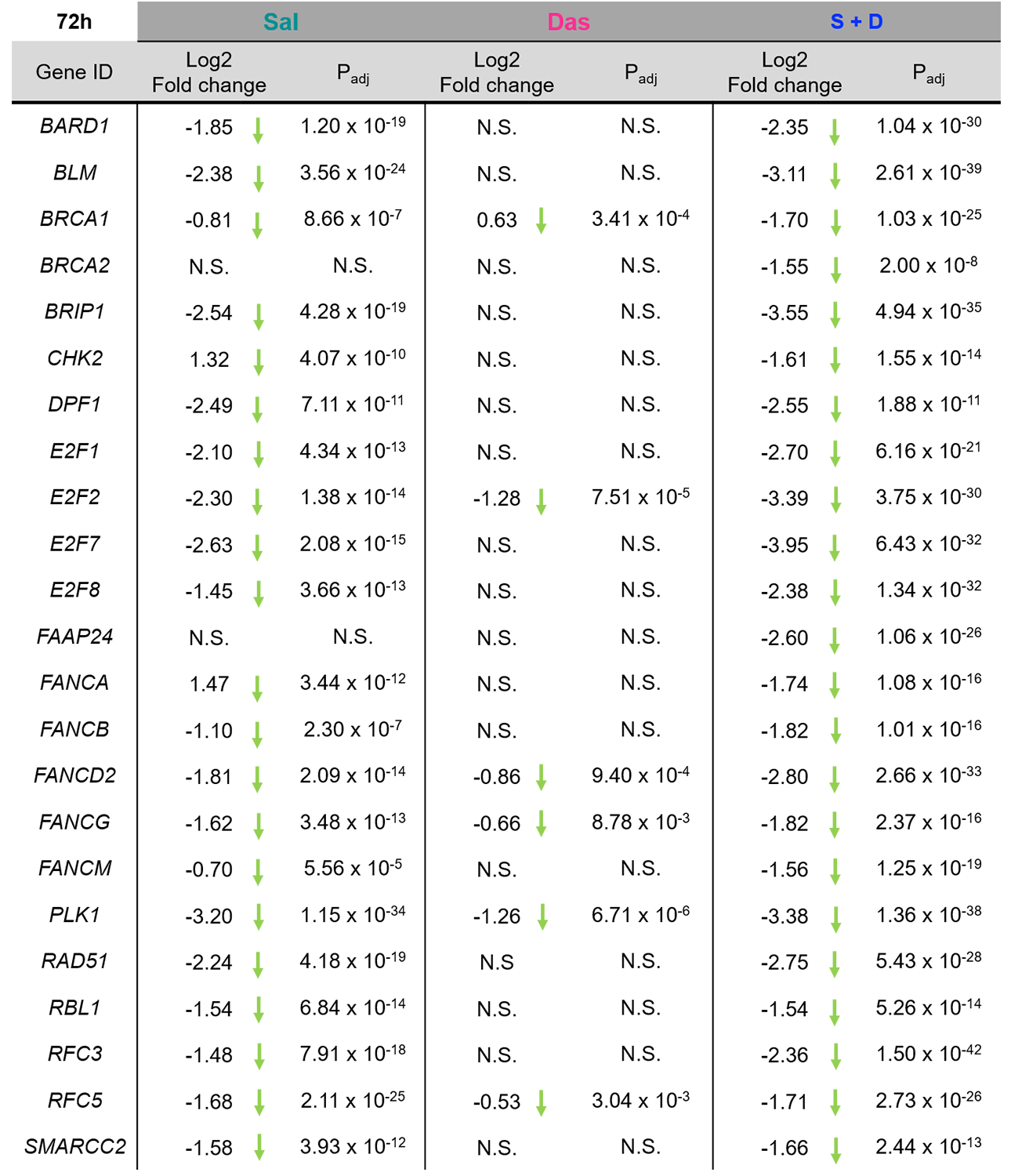

Supplement: Supplementary file 18 — Additional file 18 Figure S16: Sal and Das together synergistically inhibited the BRCA1 pathway in a MDA-MB-468 cell line. A table summarizing the differential gene expression of cells treated with Sal (0.5 μM) or Das (15 μM), or the drug combination. The experiments were performed in quadruplicate. N.S. = no significant change of the gene expression. [file 12885_2020_7134_MOESM18_ESM.tif]
